# Oncogenic role of rab escort protein 1 through EGFR and STAT3 pathway

**DOI:** 10.1038/cddis.2017.50

**Published:** 2017-02-23

**Authors:** Un-Jung Yun, Jee Young Sung, Seog-Yun Park, Sang-Kyu Ye, Jaegal Shim, Jae-Seon Lee, Masahiko Hibi, Young-Ki Bae, Yong-Nyun Kim

**Affiliations:** 1 Comparative Biomedicine Research Branch, Division of Cancer Biology, National Cancer Center, Goyang, Korea; 2 Pediatric Oncology Branch, National Cancer Center, Goyang, Korea; 3 Department of Pathology, National Cancer Center, Goyang, Korea; 4 Department of Pharmacology, College of Medicine, Seoul National University, Seoul, Korea; 5 Department of Molecular Medicine, College of Medicine, Inha University, Incheon, Korea; 6 Bioscience and Biotechnology Center, Nagoya University, Nagoya, Japan

## Abstract

Rab escort protein-1 (REP1) is linked to choroideremia (CHM), an X-linked degenerative disorder caused by mutations of the gene encoding REP1 (*CHM*). REP1 mutant zebrafish showed excessive cell death throughout the body, including the eyes, indicating that REP1 is critical for cell survival, a hallmark of cancer. In the present study, we found that REP1 is overexpressed in human tumor tissues from cervical, lung, and colorectal cancer patients, whereas it is expressed at relatively low levels in the normal tissue counterparts. REP1 expression was also elevated in A549 lung cancer cells and HT-29 colon cancer cells compared with BEAS-2B normal lung and CCD-18Co normal colon epithelial cells, respectively. Interestingly, short interfering RNA (siRNA)-mediated REP1 knockdown-induced growth inhibition of cancer cell lines via downregulation of EGFR and inactivation of STAT3, but had a negligible effect on normal cell lines. Moreover, overexpression of REP1 in BEAS-2B cells enhanced cell growth and anchorage-independent colony formation with little increase in EGFR level and STAT3 activation. Furthermore, REP1 knockdown effectively reduced tumor growth in a mouse xenograft model via EGFR downregulation and STAT3 inactivation *in vivo*. These data suggest that REP1 plays an oncogenic role, driving tumorigenicity via EGFR and STAT3 signaling, and is a potential therapeutic target to control cancers.

Epidermal growth factor receptor (EGFR) activation induces activation of various signaling pathways, including Ras-MAPK, PI3K-AKT, Src, and STAT3, which are important for EGF-mediated cell proliferation, differentiation, cell motility, and cell survival.^1, 2, 3^ EGFR expression and activation are tightly regulated during normal development and tissue homeostasis. Therefore, dysregulation of EGFR and/or hyperactivation of EGFR as a result of overexpression or mutation is tightly linked to pathogenesis of human cancers.^2^ Deletion of exons 2–7 of EGFR results in ligand-independent, constitutively active EGFR variant III, which is the most common EGFR mutation in glioblastoma.^4^ In addition, EGFR deletions in exon 19 and EGFR mutations such as L858R are found in lung cancer and are sensitive to the tyrosine kinase inhibitor gefitinib. However, the most common secondary mutation, T790M, causes resistance to gefitinib, indicating that a strategy to overcome acquired resistance is also critical to control of lung cancer.^5^

EGFR levels at the cell surface can be regulated by EGFR trafficking from the plasma membrane to the cytosol and vice versa. Ligand binding to EGFR leads to endocytosis of EGFR, and the internalized EGFR is sorted for recycling back to the cell surface or targeted for lysosomal degradation to attenuate persistent EGFR signaling.^6, 7^ Internalization-defective EGFR mutants may escape the ligand-induced lysosomal degradation pathway, leading to prolonged cell signaling and tumor development.^8^ Therefore, activation of EGFR endocytosis and downregulation of EGFR are considered to be therapeutically relevant in cancers.^7, 9^

Rab GTPases play a critical role in targeting EGFR for recycling or lysosomal degradation.^10^ The first step in the activation of Rab GTPases is geranyl-geranyl modification of Rab GTPases, a process mediated by Rab escort protein 1 (REP1) and Rab geranyl-geranyl transferase 2.^11, 12, 13^ Mutations of the gene encoding human REP1 result in the X-linked eye disease known as choroideremia (CHM), which is characterized by progressive degeneration of the retinal pigment epithelium (RPE), photoreceptors, and choroid.^14^ Knockout of the mouse gene (*Chm*) leads to malformation of photoreceptors and defects in the RPE.^15, 16^ In zebrafish *chm* mutation results in the choroideremia phenotype^17, 18^ and leads to devastating hair cells and cell death of photoreceptor as well as degeneration of the RPE.^18, 19^ In addition to eye defects, chm−/− zebrafish show early embryonic lethality with widespread cell death across the entire embryo at 5 days post fertilization (d.p.f.).^20^ These studies suggest that REP1 function is critical for cell survival. Therefore, it is conceivable that REP1 expression is linked to cell growth and/or survival, which is important for tumor development. In this study, we first demonstrate that REP1 expression is upregulated in cancer cells and cancer patient tissue and that REP1 (*CHM*) functions as an oncogene. We show that REP1 knockdown leads to cell growth inhibition and/or cell death via EGFR downregulation and STAT3 inactivation. Finally, we present evidence that REP1 knockdown inhibits growth of human tumor xenografts in mice, with downregulation of EGFR. Together, these findings identify REP1 as a potential novel therapeutic target for control of cancer.

## Results

### Upregulation of REP1 in cancer patient tissues and cancer cell lines

We have demonstrated that the phenotype of the zebrafish *eva*^rk10^ mutant, which expresses a GDP dissociation inhibitor (GDI) domain-truncated REP1 protein, is eye degeneration similar to that seen with previously reported alleles of *chm* mutants (Supplementary Figure S1a–c).^17^ It has been reported that chm−/− zebrafish undergo early embryonic lethality with apoptotic cell death in various organs at 5 d.p.f.^20^ We also observed that the zebrafish *eva*^*rk10*^ mutant was lethal at 5 d.p.f. with increased cell death in the eyes and brain as determined by TUNEL assay (Supplementary Figure S1d). Caspase 3 activation was strongly detected in eyes, tectum, and cerebellum in *eva*^*rk10*^ mutant embryos compared with wild-type embryos (Supplementary Figure S1e), suggesting that REP1 plays an important role, not only in normal development, but also in cell survival of various tissues in zebrafish embryos.

**Figure 1:**
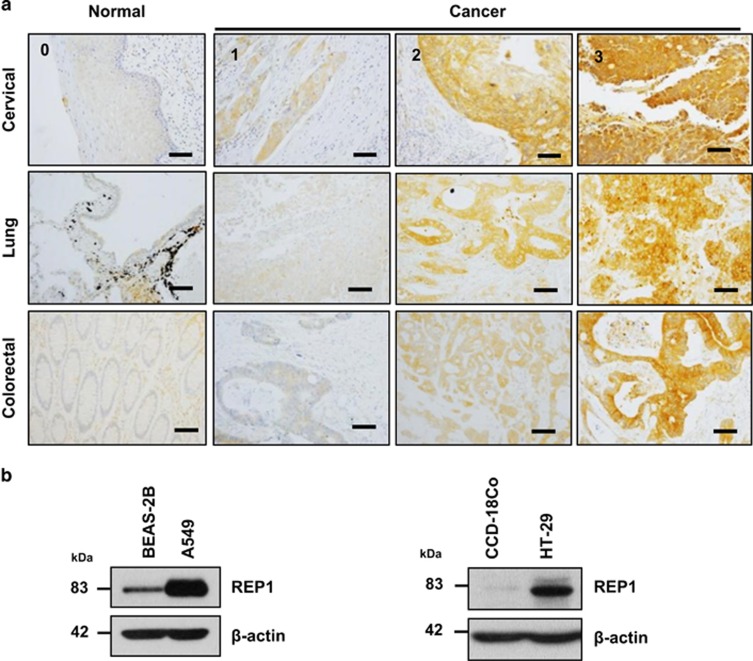
REP1 expression in human cancer tissues and cancer cell lines. (**a**) Cancer patient-derived microarrays for cervical, lung, and colorectal tissue were examined for REP1 expression using an immunoperoxidase method. Staining results were graded according to the intensity and proportion of positive cells as described in ‘Materials and Methods'. Scale bar=50 *μ*m. (**b**) A human normal lung epithelial cell line BEAS-2B and a lung adenocarcinoma cell line A549, and a normal colon epithelial cell line CCD-18Co and a colorectal adenocarcinoma cell line HT-29 were processed for immunoblot analysis using anti-REP1 antibody and *β*-actin antibody was used as a loading control. These experiments were performed three independent times with comparable results

Because REP1 mutant zebrafish showed excessive cell death in the intestine as well as in the eyes and brain (Supplementary Figure S1) and REP1 mRNA levels are elevated in several human tumor tissues,^21^ it is possible that REP1 has an oncogenic function. First, we examined REP1 expression levels using tissue microarrays (TMAs) prepared from tissue of cervical, lung, and colorectal cancer patients. Each array contained samples of normal and cancer tissue. Immunohistochemistry analysis of TMAs revealed that REP1 was expressed at a high level in all three types of cancer tissue, whereas expression was minimal in normal tissues (Figure 1a and Supplementary Figure S2). The results of TMA-based analysis of REP1 expression are shown in Table 1 and Supplementary Table S1–3. In addition, REP1 was expressed at a high level in A549 lung adenocarcinoma cells and HT-29 colon cancer cells, but weakly or rarely expressed in BEAS-2B and CCD-18Co, the normal counterparts of A549 and HT-29 cells, respectively (Figure 1b). These data indicate that REP1 is upregulated in human cancers.

### Cell growth inhibition and apoptosis with REP1 knockdown

**Figure 2:**
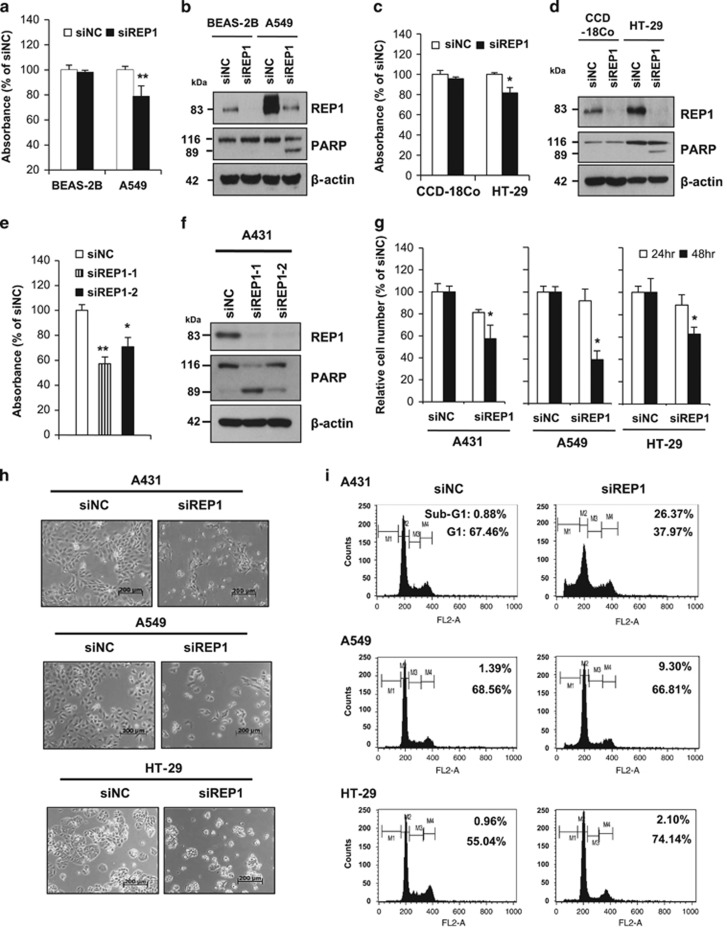
Effects of REP1 knockdown on cell growth and apoptosis. (**a** and **b**) BEAS-2B and A549 cells were transfected with either siNC or siREP1. Cell growth was measured by MTS assay at 48 h after transfection, with error bars representing S.D. (versus siNC, **P<0.01) (**a**) and cellular proteins were subjected to immunoblot analysis using indicated antibodies (**b**). (**c** and **d**) CCD-18Co and HT-29 cells were transfected with either siNC or siREP1. Cell growth was measured by MTS assay at 48 h after transfection, with error bars representing S.D. (versus siNC, *P<0.05) (**c**) and cellular proteins were subjected to immunoblot analysis using indicated antibodies (**d**). (**e**) A431 cells were transfected with either non-specific control siRNAs (siNC) or two different siRNAs specific for REP1 (siREP1-1 and siREP1-2) and cell growth was measured by MTS assay at 48 h after transfection, with error bars representing S.D. (versus siNC, *P<0.05, **P<0.01). (**f**) A431 cells were treated as in **e** and cell lysates were subjected to immunoblot analysis using anti-REP1, -PARP, and -*β*-actin antibodies. (**g**, **h** and **i**) A431, A549, and HT-29 cells were transfected with either siNC or siREP1. Live cells were counted by trypan blue staining, with error bars representing S.D. (versus siNC, *P<0.05) (**g**), cell images were taken using phase contrast microscopy. Magnification: × 50; scale bar=200 *μ*m (**h**), or cells were subjected to Sub-G1 analysis by flow cytometry (M1, sub-G1 phase; M2, G1 phase; M3, S phase; M4, G2/M phase) (**i**). Similar results were observed in three independent experiments

Next, we compared the effects of siRNA-mediated knockdown of REP1 on cell growth in normal and cancer cells. REP1 knockdown had little effect on growth of BEAS-2B normal lung epithelial cells, but reduced growth of A549 lung cancer cells, with increased cleavage of the caspase-3 substrate PARP (Figure 2a and b). The same pattern was observed in CCD-18Co normal colon cells and HT-29 colon cancer cells (Figure 2c and d). These results suggest that REP1 knockdown exerts its antiproliferative effect by inducing apoptosis and/or cell cycle delay in various cancer cells with little effect on normal cells.

To further elucidate a possible role of REP1 in cell survival, we suppressed REP1 expression using two different siRNAs targeting REP1 (siREP1-1 and siREP1-2) in A431 human epidermoid carcinoma cells. REP1 knockdown-induced cell growth inhibition (Figure 2e) with an increase in PARP cleavage (Figure 2f). Annexin V/PI apoptosis analysis revealed that REP1 knockdown increased annexin V positive cells (Supplementary Figure S3a), indicating that siREP1-mediated growth inhibition is associated with apoptosis. Because siREP1-1 had a stronger effect than siREP1-2 on cell growth inhibition, we used this siRNA in subsequent experiments. Cell growth inhibition by siRNA appeared not to be limited to A431 cells because REP1 knockdown suppressed growth of A549 and HT-29 cells as determined by cell counting and microscopic analysis (Figure 2g and h). Furthermore, cell cycle analysis revealed that the sub-G1 population (an apoptosis indicator), increased to 25.5% in A431 cells transfected with siREP1 compared with cells transfected with negative control siRNA (siNC), whereas it slightly increased in REP1 knockdown A549 cells compared to controls. In HT-29 cells, REP1 knockdown increased the G1 population and decreased the S and G2 populations, with a little increase in sub-G1 cells (Figure 2i and Supplementary Figure S3b). These data suggest that REP1 knockdown causes cell growth inhibition via apoptosis and cell cycle arrest.

### Downregulation of EGFR and inactivation of STAT3 by REP1 knockdown

**Figure 3:**
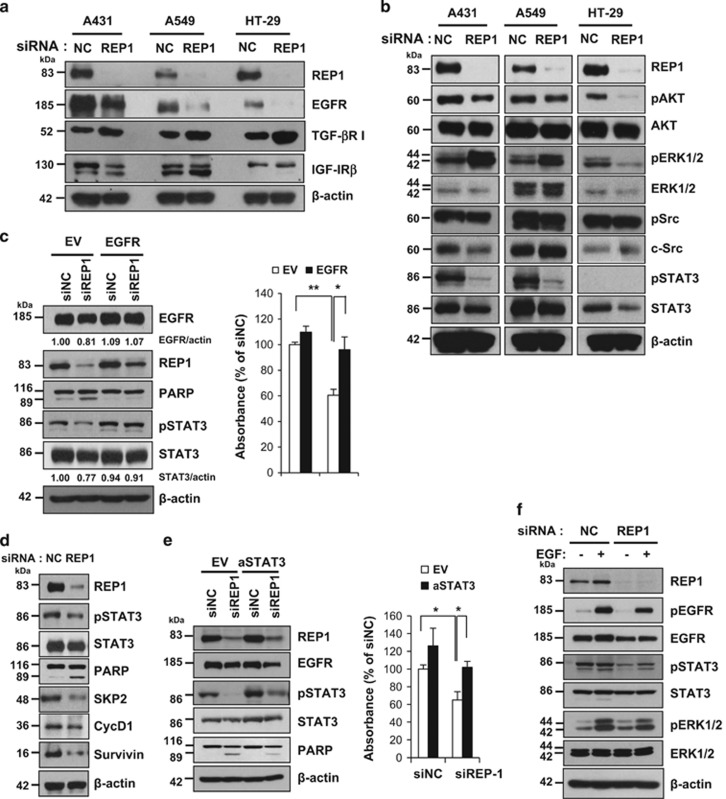
Effects of REP1 knockdown on EGFR levels. (**a** and **b**) A431, A549, and HT-29 cells were transfected with either siNC or siREP1 for 48 h and cell lysates were subjected to immunoblot analysis using indicated antibodies. (**c**) A431 cells were transfected with either empty vector (EV) and siNC, EV and siREP1, EGFR plasmid and siNC, or EGFR plasmid and siREP1 together for 48 h. Cell lysates were subjected to immunoblot analysis using indicated antibodies and cell growth was assessed by MTS assay, with error bars representing S.D. (*P<0.05, **P<0.01). (**d**) A431 cells were transfected with either siNC or siREP1 for 48 h and cell lysates were subjected to immunoblot analysis using indicated antibodies. (**e**) A431 cells were transfected with either EV and siNC, EV and siREP1, active STAT3 (aSTAT3) and siNC, or aSTAT3 and siREP1 together for 48 h. Cell lysates were processed for immunoblot analysis using indicated antibodies and cell growth was measured by MTS assay, with error bars representing S.D. (*P<0.05). (**f**) A431 cells were transfected with either siNC or siREP1 for 48 h and then treated with 1 nM EGF for 10 min. Cell lysates were subjected to immunoblot analysis using indicated antibodies. The experiments were performed three times with similar results

Growth factor receptors play a critical role in cell growth and survival, and overexpression has been associated with tumorigenesis in various cancers.^22, 23^ To investigate whether REP1 regulates levels of growth factor receptors, we examined several receptors after REP1 knockdown. TGF-*β*R I level was increased and IGF-IR*β* level remained unchanged after REP1 knockdown (Figure 3a). Although there was a little decrease in the levels of PDGFR-*α* and c-MET (Supplementary Figure S4), EGFR downregulation appeared to be marked in all three cell lines (A431, A549, and HT-29) upon REP1 knockdown (Figure 3a). Accordingly, phospho-EGFR was reduced in these three cell lines by REP1 knockdown, with an increase in PARP cleavage (Supplementary Figure S5). Because REP1 knockdown resulted in EGFR downregulation, we investigated EGFR downstream signaling pathways that are involved in cell growth. REP1 knockdown decreased AKT activation in HT-29 cells but had little effect in A431 and A549 cells. ERK1/2 activation was rather increased in A431 and A549 cells but decreased in HT-29 cells with REP1 knockdown. There was little change in Src activation in all three cell lines with REP1 knockdown; however, STAT3 activation was markedly reduced (Figure 3b and Supplementary Figure S5).

Next, we tested whether EGFR overexpression attenuates inhibition of cell growth induced by REP1 knockdown in A431 cells. Overexpression of exogenous EGFR increased REP1 levels and STAT3 activation initially. REP1 knockdown-mediated downregulation of EGFR and inactivation of STAT3 were alleviated and, thus, REP1 knockdown-induced cell death was reversed together with reduction of PARP cleavage in the cells overexpressing EGFR (Figure 3c and Supplementary Figure S6a). REP1 protein levels remained higher in the EGFR overexpressing cells than in the EV cells upon siREP1 treatment. It is probable that the apparent rescue of REP1 knockdown-induced cell growth inhibition, increased apoptosis and decreased STAT3 activation by EGFR overexpression could be mediated by a much weaker REP1 downregulation due to the persistence of the REP1 protein in the presence of EGFR overexpression. Because REP1 knockdown inhibited STAT3 activation, we examined STAT3 target genes involved in cell growth and survival, including SKP2, cyclin D1, and survivin.^24, 25, 26^ REP1 knockdown decreased protein levels of SKP2, cyclin D1, and survivin in A431 and A549 cells (Figure 3d and Supplementary Figure S6b). Overexpression of active STAT3 attenuated PARP cleavage and cell death induced by si-REP1 (Figure 3e). Because REP1 knockdown results in EGFR downregulation, we speculated that REP1 knockdown cells would be less responsive to EGF stimulation. As expected, EGF-stimulated EGFR activation was alleviated and, thus, activation of STAT3 and ERK1/2 were decreased in the REP1 knockdown cells compared with siNC-transfected cells (Figure 3f). Treatment of A431 cells with an EGFR inhibitor, AG1478, decreased cell growth (Supplementary Figure S7a), indicating that EGFR activity is important for A431 cell growth. In addition, knockdown of either EGFR or STAT3 inhibited cell growth but REP1 knockdown was most effective for cell growth inhibition (Supplementary Figure S7b and c). All these data indicate that REP1 knockdown inactivates EGFR and STAT3, thereby inducing growth inhibition and/or cell death.

### Oncogenic effects of REP1

**Figure 4:**
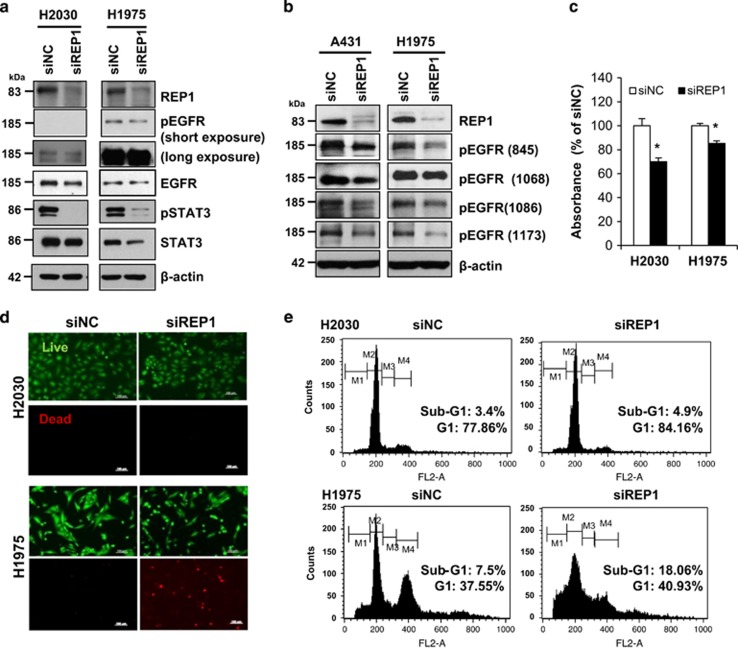
Effects of REP1 knockdown on NSCLC with EGFR mutation. (**a**) H2030 and H1975 cells were transfected with either siNC or siREP1 for 48 h and cell lysates were subjected to immunoblot analysis using indicated antibodies. (**b**) A431 and H1975 cells were transfected with either siNC or siREP1 for 48 h and cell lysates were processed for immunoblot analysis using anti-phospho-EGFR antibodies specific for tyrosine residues at 845, 1068, 1086, and 1173, respectively. (**c**, **d** and **e**) H2030 and H1975 cells were transfected with siRNAs as in **a**, followed by MTS assay, with error bars representing S.D. (*P<0.05) (**c**), live/dead analysis by staining with calcein-AM and PI (**d**), and cell cycle analysis by flow cytometry (**e**). Images were taken by fluorescence microscopy. Magnification: x100; Scale bar=100 *μ*m. (M1, sub-G1 phase; M2, G1 phase; M3, S phase; M4, G2/M phase). Similar results were observed in three independent experiments

The association of mutation-induced hyperactivation of EGFR with malignant lung cancer is well established.^5^ Therefore, we investigated the effects of REP1 knockdown on cell growth in other NSCLC cell lines, including H2030 cells and H1975 cells, expressing wild-type EGFR and mutant EGFR (L858R and T790M), respectively. Basal EGFR activity was very low in H2030 cells, but extremely high in H1975 cells, although H1975 cells expressed EGFR at a much lower level than H2030 cells (Figure 4a). With REP1 knockdown, there was a small decrease in EGFR level and activity, but prominent STAT3 inactivation, in both cell lines (Figure 4a). A431 and H1975 cells exhibit higher EGFR activity as a result of EGFR overexpression and mutation, respectively, and we compared changes in EGFR phosphorylation after REP1 knockdown. EGFR phosphorylation at multiple tyrosine residues, including Y845, Y1068, Y1086, and Y1173, was decreased in the REP1 knockdown cells (Figure 4b), indicating that REP1 knockdown reduced overall EGFR tyrosine phosphorylation. Next, we investigated whether REP1 knockdown induces growth inhibition differentially depending on EGFR activity. REP1 knockdown inhibited growth of both H2030 and H1975 cells (Figure 4c) but the cause of growth inhibition appeared to be different in each cell line. As determined by live/dead assay, there were fewer dead H2030 cells, but more dead H1975 cells (Figure 4d). In addition, REP1 knockdown-induced cell cycle arrest at G1 phase significantly in H2030 cells rather than in H1975 cells, but increased the sub-G1 population in H1975 cells (Figure 4e and Supplementary Figure S8). These data indicate that REP1 knockdown induces growth inhibition either by cell cycle arrest or cell death depending on EGFR activity or cell type.

**Figure 5:**
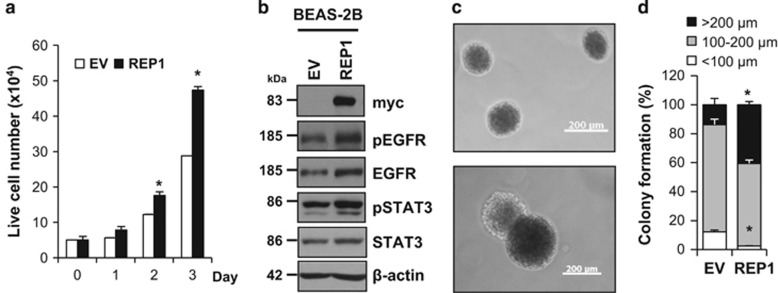
Effects of REP1 overexpression on normal cell growth. (**a** and **b**) BEAS-2B cells were transfected with either EV or REP1-myc plasmid. To assess cell growth, transfected cells were stained with trypan blue and trypan blue negative cells were counted as live cells, with error bars representing S.D. (versus EV, *P<0.05) (**a**). Cell lysates at 72 h were subjected to immunoblot analysis using indicated antibodies (**b**). (**c** and **d**) BEAS-2B cells transfected empty vector or REP1-myc plasmid were subjected to soft agar colony-formation assay. Representative colony images were taken using phase contrast microscopy. Magnification: × 50, Scale bar=200 *μ*m (**c**). Colony sizes were measured and summarized in the histogram, with error bars representing S.D. (versus EV, *P<0.05) (**d**). Similar results were observed in three independent experiments

To further investigate oncogenic effects of REP1, we overexpressed REP1 in BEAS-2B cells, a normal lung epithelial cell line. REP1 overexpression enhanced growth of BEAS-2B cells with a slight increase in EGFR level and STAT3 activation (Figure 5a and b). The ability to form colonies in soft agar, a phenotype of transformed tumor cells,^27^ was greater in the REP1-overexpressing BEAS-2B cells than in control vector-transfected BEAS-2B cells (Figure 5c and d). In addition, the REP1-mediated cell growth was reversed by either siEGFR or siSTAT3 treatment in A431 cells. However, siSTAT3, but not siEGFR, could attenuate the REP1-mediated cell growth in BEAS-2B cell (Supplementary Figure S9a–d). To test whether EGFR downregulation is important for cell growth inhibition induced by siREP1, we employed B82L, a normal mouse fibroblast cell line that lacks the EGFR.^28^ REP1 knockdown did not significantly reduce cell growth in this cell line, indicating that cell growth inhibition by REP1 knockdown is associated with EGFR (Supplementary Figure S10). All these data indicate that REP1 overexpression serves an oncogenic function via augmentation of cell growth through EGFR and STAT3.

### Antitumor effects of REP1 knockdown *in vivo* via EGFR downregulation and STAT3 inactivation

**Figure 6:**
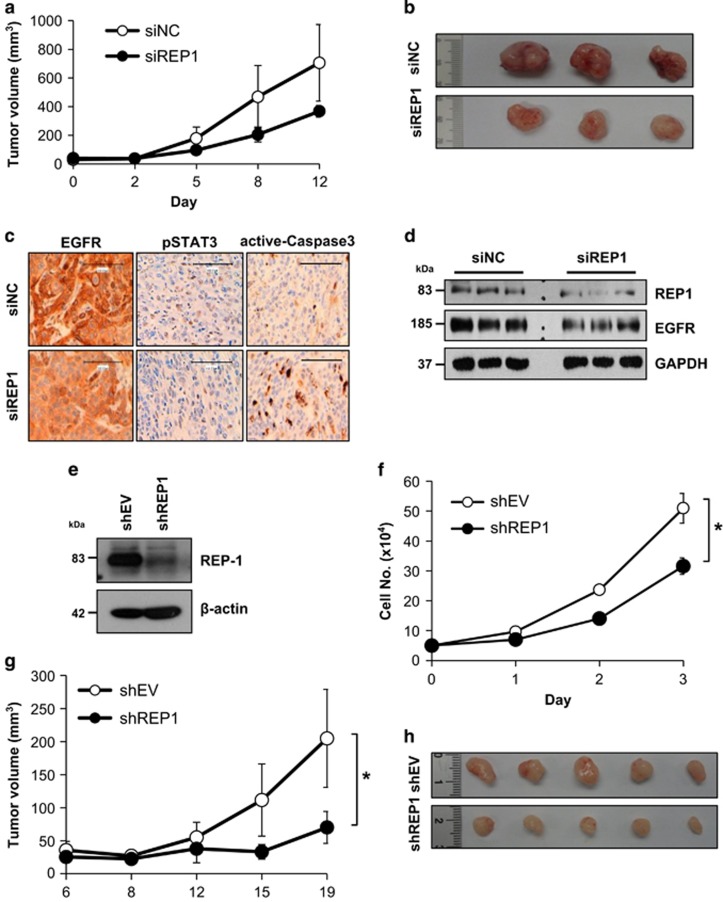
Effects of REP1 knockdown on tumor growth in the xenografted mice. (**a**, **b**, **c**, and **d**) A431 cells (2.5 × 10^6^) were injected subcutaneously into the nude mice. When tumor size reached 30 mm^3^, siRNA in the atelogene gel was injected to encompass the whole tumor mass (*n*=3 per group). Tumor size was then measured at the indicated times (**a**). After sacrificing the mice, tumor tissues were taken and representative images of tumors are shown (**b**). Tumor tissue were either stained with indicated antibodies for immunochemistry analysis (**c**) or processed for immunoblot analysis using indicated antibodies (**d**). Magnification: × 200, Scale bar=100 *μ*m. (**e**) Stable A431 cells expressing either shEV or shREP1 were established and cell lysates were processed for immunoblot analysis using indicated antibodies. (**f**) A431 cells (5 × 10^4^) expressing shEV or shREP1 were grown for 3 days and cell growth was measured by trypan blue staining at indicated times, with error bars representing S.D. (*P<0.05). (**g** and **h**) A431 cells (2.5 × 10^6^) expressing shEV or shREP1 were injected subcutaneously into the nude mice (*n*=5 per group). Tumor size was then measured at the indicated times, with error bars representing S.D. (*P<0.05) (**g**). After sacrificing the mice, tumor tissues were taken and representative images of tumors are shown (**h**). Similar results were observed in two independent experiments

To test whether REP1 knockdown has an anticancer effect, xenografts were generated in nude mice by injection of A431 cells and siRNA mixture was injected into the tumor mass using an *in vivo* siRNA delivery system. The growth of siREP1-treated tumors was significantly retarded compared with that of siNC-treated tumors (Figure 6a). When the tumors were removed from the sacrifice mice, siREP1-treated tumors were smaller than the siNC-treated tumors (Figure 6b). Although *P*-value was 0.126, we could see the tendency that siREP1 treatment could inhibit tumor growth. In addition, immunohistochemical analysis showed that EGFR levels and STAT3 activation were reduced, but active caspase-3 increased, in the siREP1-treated tumors compared with the siNC-treated tumors (Figure 6c). Immunoblot analysis revealed that both REP1 and EGFR were downregulated in the siREP1-treated tumors (Figure 6d). To investigate the effects of permanent knockdown of REP1, we established A431 cells expressing shREP1 (Figure 6e). Consistent with the data in the Figure 3a, shREP1 cells grew slower than shEV cells and tumor growth was slower in the shREP1 xenografted mice than in the shEV xenografted mice (Figure 6f–h). Furthermore, EGFR levels decreased in the shREP1 tumor compared with the shEV tumor (Supplementary Figure S11). Finally, EGFR immunoreactivity was weak overall and strong in pharyngeal arch and esophagus epithelium of wild-type zebrafish but was markedly decreased in zebrafish *eva*^*rk10*^ mutant embryos at 5 d.p.f. EGFR levels decreased in the lysates of whole zebrafish *eva*^*rk10*^ mutant embryos compared with those of wild-type embryos (Supplementary Figure S12a–c). Collectively, these data indicate that REP1 exerts its tumorigenic effects via EGFR and/or STAT3 pathway. Therefore, targeting of REP1 may be a good strategy to control tumors that exhibit a high level of EGFR activity and STAT3 activation.

## Discussion

The gene that encodes REP1 was first identified as a critical gene in choroideremia, an X-linked recessive disease of the retina that results in progressive degeneration of the retina, the RPE, and the choroid.^29^ Choroideremia results from defects in the gene that encodes REP1 in humans (*CHM*), mice (*Chm*), and zebrafish (*chm*).^19, 30, 31, 32^ In addition to the eye defect, *chm*^−/−^ mutant zebrafish show embryonic lethality at 5 d.p.f. with cell death throughout the body^20^ and, we also discovered that *eva*^*rk10*^mutant zebrafish show aberrant development with cell death (Supplementary Figure S1). In studies of mice, *Chm* has been shown to be essential for diploid trophoblast development and vascularization in mouse extraembryonic tissues.^31^ These data indicate that REP1 expression is critical not only for development but also for overall cell survival. However, REP1 function in regulation of cell survival has rarely been investigated. In our study, we first demonstrated a novel association of REP1 expression with cancer. We found that REP1 is upregulated in human cancers, including cervical, lung, and colorectal cancer, compared with their normal tissue counterparts. We also showed that REP1 knockdown leads to inhibition of cell growth and/or increased cell death via EGFR downregulation and STAT3 inactivation both *in vitro* and *in vivo*.

Interestingly, REP1 is upregulated in tumor tissue from patients with various types of cancer and in cancer cell lines whereas expression is minimal in the normal tissues and in normal cell lines we tested (Figure 1). REP1 overexpression in the normal epithelial cell line BEAS-2B enhanced cell proliferation and anchorage-independent growth in soft agar, a phenotype of transformed tumor cells (Figure 5a and c), suggesting that REP1 upregulation is associated with tumorigenesis. Furthermore, siRNA-mediated REP1 knockdown caused growth inhibition of cancer cells, but not normal cells, suggesting that REP1 expression level is more important for cancer cell survival than for survival of normal cells. It has been reported that REP1 expression is negatively associated with overall survival rates for gastric cancer patients, as illustrated by analysis of publicly available data using the Kaplan–Meier Plotter (http://kmplot.com; Affy ID: 207099_s-at, Gene symbol: CHM; total patient number=876; *P*-value=0.00033; Supplementary Figure S13a). REP1 knockdown also increased PARP cleavage in the gastric cancer cell lines (Supplementary Figure S13b). These data suggest that REP1 expression levels are linked to tumorigenesis.

REP1 knockdown resulted in downregulation of EGFR, which leads to a decrease in EGFR activation unlike other growth factor receptors, including IGF-IR*β* and TGF-*β*R I (Figure 3a). Amplification of the *EGFR* gene and mutations of the EGFR tyrosine kinase domain, which leads to EGFR hyperactivity, have been demonstrated to occur in various solid tumors.^33^ It is of interest that REP1 knockdown downregulates, and thus inactivates EGFR because EGFR inactivation is a critical strategy to control cancer. EGFR levels were also decreased in the *eva*^*rk10*^mutant zebrafish compared with wild-type zebrafish (Supplementary Figure S12).

EGFR activates signaling pathways that regulate cell growth, including RAS/RAF/MAP kinase, PI3K-AKT, Src, and STAT3 signaling pathways.^34, 35, 36^ Unlike A431 cells, A549 cells express oncogenic KRAS (G12S) and HT-29 cells express oncogenic BRAF (V600E) and PI3KCA mutants, which could bypass EGFR signaling such as RAS/RAF/MAP kinase and PI3K-AKT pathways.^37^ However, REP1 knockdown inhibited growth of all three cell lines regardless of these mutations. REP1 knockdown caused inactivation of AKT, Src, and STAT3 in all three cell lines and ERK1/2 activation was somewhat increased in A431 and A549 cells, but decreased in HT-29 cells by REP1 knockdown (Figure 3b). REP1 knockdown inactivated STAT3 strikingly in A431 and A549 cells and somewhat decreased STAT3 levels in HT-29 cells (Figure 3b and Supplementary Figure S6). It is likely that EGFR downregulation by siREP1 results in STAT3 inactivation, which is responsible for cell growth inhibition regardless of bypassing mutants such as KRAS (A549) and BRAF/PI3KCA (HT-29). STAT3 is activated in response to growth factors and cytokines, and induces expression of target genes, including SKP2, cyclin D1, c-Myc, survivin, and Bcl-xL, which are associated with cell proliferation and survival.^26, 38, 39, 40^ REP1 knockdown inactivated STAT3 and, consequently, levels of SKP2, cyclin D1, and survivin were decreased (Figure 3d). EGFR overexpression attenuated cell death and maintained STAT3 activation in REP1 knockdown cells (Figure 3c). Active STAT3 expression also effectively rescued cells from REP1 knockdown-induced cell death (Figure 3e). These findings strongly suggest that REP1 expression is linked to the EGFR/STAT3 signaling pathway.

There was no significant difference in *EGFR* mRNA levels in control siNC- and siREP1-treated cells, indicating that EGFR levels are not regulated at the transcriptional level (Supplementary Figure S14a). The lysosome inhibitor chloroquine, but not the proteasome inhibitor MG132, slightly reversed EGFR downregulation in the REP1 knockdown cells (Supplementary Figure S14b and c), indicating that EGFR trafficking might be altered by siREP1 treatment. In addition, REP1 knockdown did not increase EGFR phosphorylation at tyrosine 1045, the binding residue of the major c-Cbl, thereby mediating EGFR ubiquitination and degradation,^41^ indicating independency of the proteosomal pathway (Supplementary Figure S15). It is possible that REP1 regulates EGFR signaling positively either by favoring recycling, inhibiting lysosome degradation or favoring active signaling from endosomes. EGFR downregulation and degradation are regulated mainly by endocytic trafficking. Ligand-bound EGFR accelerates clathrin-mediated internalization, followed by the efficient lysosomal targeting of internalized receptors.^6^ Rab small G proteins are master regulators of intracellular vesicle trafficking^42^ and several Rabs have a crucial role in EGFR trafficking.^43^ Rab5 mediates EGFR entry into the early endosome and Rab11 facilitates EGFR recycling to the plasma membrane. Rab7 is required for lysosomal degradation of EGFR.^44^ REP1 is important for geranyl-geranyl modification of Rab GTPases, which is the first step in Rab GTPase activation.^13^ In our study, REP1 knockdown increased Rab5 levels but decreased Rab7 levels, with little change in Rab11 levels (Supplementary Figure S16). It has been reported that Rab5A depletion in cancer cells delays EGFR degradation, but Rab5A overexpression increases EGFR degradation via acceleration of EGFR trafficking.^45^ Rab7 knockdown blocks constitutive recycling of non-ligand-bound EGFR to the cell surface due to accumulation in the late endosome.^46^ It is probable that increased Rab5 and decreased Rab7 levels might block EGFR recycling but enhance EGFR degradation in the REP1 knockdown cells. However, we cannot rule out the possibility that other Rab proteins are involved in EGFR downregulation induced by REP1 knockdown, which remains to be elucidated.

In summary, REP1 is upregulated in several types of cancer tissue with minimal expression in the normal tissue counterparts. REP1 knockdown induces cell growth inhibition in cancer cells, but not normal cells, via downregulation and inactivation of EGFR and STAT3, respectively. In addition, REP1 knockdown showed antitumor effects in a human tumor xenograft mouse model with EGFR downregulation. Taken together, our data suggest that targeting of REP1 may be a new therapeutic strategy to control tumor growth.

## Materials and methods

### Materials

Anti-REP1, anti-EGFR, anti-c-Src, anti-IGF-1R*β*, horseradish peroxidase (HRP)-conjugated anti-rabbit IgG, and HRP-conjugated anti-mouse IgG antibodies were purchased from Santa Cruz Biotechnology (Santa Cruz, CA, USA). Phospho-EGFR antibodies (Y845, Y1068, Y1086, and Y1173) were obtained from Invitrogen (Eugene, OR, USA). Anti-phospho-Src (Y416), anti-STAT3, anti-phospho-STAT3 (Y705), anti-TGF-*β* receptor I, anti-phospho-AKT (T308), cleaved caspase 3, SKP2, Survivin, Cyclin D1, ERK1/2, phospho-ERK1/2, and anti-PARP antibodies were purchased from Cell Signaling Technology (Beverly, MA, USA). Anti-GAPDH was purchased from Abfrontier (Seoul, Korea). Anti-*β*-actin antibody was purchased from Sigma-Aldrich Corporation (St. Louis, MO, USA).

### Cell culture

Human epidermoid carcinoma cell line A431, human lung cancer cell line A549, non-small-cell lung cancer (NSCLC) cell lines H2030 and H1975, and human colon cancer cell line HT-29 were obtained from the American Type Culture Collection (ATCC, Rockville, MD, USA). Human normal lung epithelial cell line BEAS-2B was obtained from the ATCC and a human normal colon epithelial cell line CCD-18Co was obtained from the Korean Cell Line Bank (KCLB, Seoul, Korea). A431 and CCD-18Co cells were grown in DMEM (Hyclone, Logan, UT, USA) supplemented with 10% FBS (Hyclone, Logan, UT, USA). BEAS-2B cells were grown in Keratinocyte-SFM containing 0.2 ng/ml epidermal growth factor (EGF) and 30 *μ*g/ml bovine pituitary extract (BPE). A549, HT-29, H2030, and H1975 cells were grown in RPMI supplemented with 10% FBS, 100 units/ml penicillin, 100 *μ*g/ml streptomycin, and 0.25 *μ*g/ml amphotericin B (Antibiotic-Antimycotic, Gibco Laboratories Co., Grand Island, NY, USA) at 37 °C in a humidified atmosphere containing 5% CO_2_.

### Plasmids, siRNAs, and transfection

For the construction of human full length REP1, *CHM* gene were amplified from Hela cell cDNA using primers (5′-CCATCGATTATGGCGGATACTCTCCCTTCG-3′ and 5′-GTAGGCGCGCCTTCAGAGGACTCCTCTAGGTT-3′). The PCR products were myc tagged and inserted into the Cla1 and Asc1 site of pCS4+ vectors, kindly provided by Dr. Chang-Yeol Yeo (Ewha Womans University, Korea). Reverse transfection of siRNA duplexes into cells was performed using lipofectamine RNAimax (Invitrogen, Carlsbad, CA, USA) and transfection of plasmid into cells was performed using lipofectamine 2000 (Invitrogen, Carlsbad, CA, USA) as described by the manufacturer. Sequence of siRNA for the negative control (NC) was 5′-CCUACGCCACCAAUUUCGU-3′ (Bioneer, Korea). Sequences for REP1 #1 and #2 were 5′-CCGGAGAGUUCUGCAUGUU-3′ and 5′-GCAUGAAAGGCACCUAUUU-3′, respectively.

### Establishment of the stable cell line expressing shRNA for REP1 (shREP1)

REP1 shRNA duplex were cloned into pLKO.1 puro vector digested with Age1 and EcoR1. The oligonucleotide sequence of shRNA for REP1 is 5′-CCGGTCCGGCGGTATGGCAACACTCCATTTCTCGAGAAATGGAGTGTTGCCATACCGTTTTTG-3′. Lentiviral particle production and delivery to cells were performed according to previous reports.^47^ A431 cells were transduced overnight with supernatant containing lentivirus, supplemented 8 *μ*g/ml polybrene and selected with 2 *μ*g/ml puromycin.

### Cell viability and proliferation assay

Cells were collected using accutase (Innovative cell technologies, San diego, CA) and then stained with trypan blue solution. Viable cells, trypan blue negative, were counted using hemocytometer under microscope. Proliferation was determined with celltiter 96 aqueous nonradioactive cell proliferation assay kit (MTS; 3-(4,5-dimethylthiazol-2-yl)-5-(3-carboxyme-thoxyphenyl)-2-(4-sulfophenyl)-2H-tetrazolium, Promega, Madison, WI, USA) as described in the manufacturer's instruction. Absorbance was measured at 490 nm with a powerwave HT spectrophotometer (Biotek instruments, Winooski, VT, USA).

### Immunoblot analysis

Cells were lysed with 2 × sodium dodecyl sulfate-polyacrylamide gel electrophoresis (SDS-PAGE) lysis buffer (20 mM Tris, pH 8.0, 2% SDS, 2 mM dithiothreitol (DTT), 1 mM Na_3_VO_4_, 2 mM ethylenediaminetetraacetic acid (EDTA), and 20% glycerol) and sonicated. Equal amounts of protein were separated by SDS-PAGE and transferred to polyvinylidene difluoride (PVDF) membranes. The membranes were then incubated with the primary antibody at 4 °C overnight and incubated with HRP-conjugated goat anti-mouse and -rabbit IgG secondary antibodies for 1 h at room temperature. The immune complexes were visualized using chemiluminescence method. Densitometry analysis of immunoblotting images was performed using Image J software (NIH, Bethesda, MD, USA).

### Flow cytometry analysis

Cells were harvested, fixed with 70% ethanol, and stained with PI staining solution (20 *μ*g/ml PI, 0.1% Sodium citrate, 50 *μ*g/ml RNase A, 0.03% NP-40, PBS), and then analyzed by flow cytometry. The data were analyzed with Cell Quest Software (BD Bioscience, San Jose, CA, USA).

### Immunohistochemical staining for cancer tissue microarray

Tissue arrays were obtained from Superbiochips Laboratories (Seoul, Korea) as described previously.^48^ Each slide contained section of tumor and normal tissues obtained from cancer patient by biopsy or surgical resection. The tissues incubated with primary antibody for 1 h were then treated with anti-mouse biotinylated antibody (Vector Laboratories, Burlingame, CA, USA) for 1 h. Color reaction was developed by incubation with diaminobenzidine solution (Sigma-Aldrich, St. Louis, MO, USA) followed by counter staining with hematoxylin. Parallel sections incubated with normal IgGs instead of primary antibodies were used as negative controls. The overall staining results were scored from 0 to 3 based on the intensity and positive rate of staining. Intensity of staining was categorized as 0, negative; 1, weak; 2, intermediate; 3, strong. Stained tissue arrays were reviewed by experienced pathologists.

### Establishment of xenograft and tumor siRNA transfection *in vivo*

A431 cells (2.5 × 10^6^) was injected subcutaneously into 6-week-old Balb/c nude mice. Average tumor volume was determined as (*L* × *W*^2^)/2 with measurements of tumor length (*L*) and width (*W*) taken with a caliper. When the tumor reached an average volume of 30 mm^3^, siRNA mixtures were injected using atelogenes local use (Koken, Japan).^49^ Tumor volumes were determined twice a week after the siRNA gel injection. A431 cells (2.5 × 10^6^) expressing shEV or shREP1 were injected subcutaneously into Balb/c nude mice and tumor volumes were determined twice a week. Finally, the mice were killed and tumor tissues were either processed for immunoblotting analysis or immunohistochemistry analysis. For immunohistochemistry, tumor tissues were fixed with 10% neutral buffered formalin. Formaldehyde fixed specimens were paraffin-embedded and cut at a thickness of 4 *μ*m. Sections were dried for 1 h at 56 °C and immunohistochemical staining was performed with the automated instrument Discovery XT (Ventana medical systems, Tucson, AZ, USA) as follows: sections were deparaffinized, rehydrated with EZ prep (Ventana medical systems), and washed with reaction buffer (Ventana medical systems). The antigens were retrieved with heat treatment in Tris–EDTA buffer (CC1, Ventana medical systems) at 90 °C for 30 min with indicated antibodies.

### Soft agar colony-formation assay

Six-well plates were coated with a base layer of 2 ml 0.9% agarose and cells were seeded at a density of 2000 cells per well in media containing 0.54% agarose. Cells were incubated for up to 22 days and media were added every 3 to 4 days. Colonies were size-determined and counted using a light microscope.

### Statistical analysis

All data points represented the mean value of experiments in triplicates. Statistical significance was determined by Student's two tailed *t*-test, with *P*<0.05 taken to show significant differences between means.

## Figures and Tables

**Table 1:** Summary of REP1-IHC in normal and tumor tissues within the TMAs

**Organ**	**REP1 expression**		**Total (*N*)**	***P*****-value**
	**−/+ *N* (%)**	**++/+++ *N* (%)**		
*Cervical*				
Normal	4 (100.0%)	0 (0.0%)	4	0.0000
Cancer	6 (12.0%)	44 (88.0%)	50	
				
*Lung*				
Normal	9 (100%)	0 (0.0%)	9	0.0000
Cancer	12 (30.0%)	28 (70.0%)	40	
				
*Colorectal*				
Normal	8 (88.9%)	1 (11.1%)	9	0.0002
Cancer	10 (25%)	30 (75%)	40	
