# Supplementary material for: Oncogenic role of rab escort protein 1 through EGFR and STAT3 pathway
Source: Cell Death Dis. 2017 Feb 23;8(2):e2621–. doi: 10.1038/cddis.2017.50 (PMC5386492; doi:10.1038/cddis.2017.50)
Supplement: Supplementary Information [file cddis201750x1.docx]

**Materials and Methods**

**Materials** Anti-REP1, anti-EGFR, anti-PDGFR-α, anti-c-Met, anti-Rab5, anti-Rab7, anti-Rab11, Phospho-EGFR antibodies (Y1045), horseradish peroxidase (HRP)-conjugated rabbit IgG, and HRP-conjugated mouse IgG were purchased from Santa Cruz Biotechnology (Santa Cruz, CA). Phospho-EGFR (Y1068) antibody was obtained from Invitrogen (Eugene, Oregon). Anti-STAT3, anti-phospho-STAT3 (Y705), SKP2, Survivin, Cyclin D1, and anti-PARP antibodies were purchased from Cell Signaling Technology (Beverly, MA). Anti-HIF-1α antibody was kind gift from Dr. Jong-Wan Park (Seoul National University, Seoul, Korea). Anti-β-actin antibody, chloroquine, and MG132 were purchased from Sigma-Aldrich Corporation (St. Louis, MO).

**Cell culture** Human epidermoid carcinoma cell line A431, human lung cancer cell line A549, and gastric cancer cell lines AGS were obtained from the American Type Culture Collection (ATCC, Rockville, MD). SNU638 cells were obtained from the Korean Cell Line Bank (KCLB, Seoul, Korea). A431 cells were grown in DMEM (Hyclone, Logan, UT) supplemented with 10% FBS (Hyclone, Logan, UT) and AGS cells were grown in F-12K supplemented with 10% FBS. A549 and SNU638 cells were grown in RPMI with L-glutamine (Hyclone, Logan, UT) supplemented with 10% FBS, 100 units/ml penicillin, 100 μg/ml streptomycin, and 0.25 μg/ml amphotericin B (Antibiotic-Antimycotic, Gibco Laboratories Co., Grand Island, NY) at 37°C in a humidified atmosphrere containing 5% CO_2_.

**Plasmids, siRNAs and transfection** For the construction of human full length REP1, *CHM* gene were amplified from Hela cell cDNA using primers (5’- CCATCGATTATGGCGGATACTCTCCCTTCG-3’ and 5’ –GTAGGCGCGCCTTCAGAGGACTCCTCTAGGTT-3’). The PCR products were myc tagged and inserted into the Cla1 and Asc1 site of pCS4+ vectors, kindly provided by Dr. Chang-Yeol Yeo (Ewha Womans University, Korea). Reverse transfection of siRNA duplexes into cells was performed using lipofectamine RNAimax (Invitrogen, Carlsbad, CA) and transfection of plasmid into cells was reversely performed using lipofectamine 2000 (Invitrogen, Carlsbad, CA) as described by the manufacturer. Sequence of siRNA for the negative control (NC) was 5’-CCUACGCCACCAAUUUCGU-3’ (Bioneer, Korea). Sequences of siRNA for REP1-1 and -2 were 5’-CCGGAGAGUUCUGCAUGUU-3’ and 5’-GCAUGAAAGGCACCUAUUU -3’, respectively. The sequence of siRNA for EGFR and STAT3 was 5’- GAUCCACAGGAACUGGAUA-3’ (Bioneer, Korea) and 5’- CCUGCAAGAGUCGAAUGUUCUCUAU-3’, respectively.[^1^](#_ENREF_1) The sequence of siRNA for Rab7-1 and -2 were 5’-GUCUAGUUCCCUUCUGUGU-3’ and 5’-ACAGAUCUGUCCUAGUGAU-3’ respectively (Bioneer, Korea). The sequence of siRNA for mouse REP1-1 and -2 were 5’-GGAUUUGCACAAAGAUGUU-3’ and 5’-CCAUCCCGAUGAGUACAAA-3’, respectively.

**Maintenance, immunostaining, and TUNEL assay** **of zebrafish** Wild-type (WT) zebrafish (*danio rerio*) and *evanescence (eva^rk10^)* mutant zebrafish were maintained in accordance with accepted standard procedures approved by the IACUC at National Cancer Center in Korea.^2^ For positional cloning, the *eva^rk10^* heterozygous mutants were mated with wild-type India or TL zebrafish to generate F1 families. The F2 generation homozygous *eva^rk10^* mutant embryos were selected by morphological criteria and their genomic DNA was used for segregation analysis with SSLP (Simple sequence length polymorphism) markers as described previously.[^3^](#_ENREF_1) For whole mount histological analysis, zebrafish embryos were incubated with 0.006% phenylthiourea at 12 hpf (hours post fertilization) to prevent pigmentation until 5 dpf (days post fertilization). After fixation in 4% paraformaldehyde (PFA) overnight at 4°C, the embryos were washed three times with PBS and followed by 100% methanol immersion at -20°C. The embryos were then rehydrated and treated with proteinase K (10 μg/mL) for 30 min at room temperature. TUNEL assays were performed with DeadEnd™ Colorimetric TUNEL System (G7360, Promega) according to manufacturer’s instructions. For immunostaining of sections, zebrafish embryos were fixed in 4% PFA overnight at 4°C, and rehydrated progressively (75%, 50%, 25%, and 0% of methanol in PBS). The specimens were immersed progressively (5%, 10%, 20%, and 30% of sucrose in PBS) at 4 °C, frozen in OCT compound (Tissue-Tek^®^, Sakura Finetek), and sectioned at 12–14 μm with a cryostat. Sections were immunostained using anti-EGFR (sc-03, Santa Cruz) and anti-cleaved caspase 3 (D175, Cell Signaling Technology) antibodies as described previously.[^4^](#_ENREF_1) The secondary antibodies for visualization with fluorescence were used with Alexa Fluor 555 goat-anti-rabbit antibody (A-21428, Molecular Probes, Eugene, OR).  The nuclei were counterstained with hoechst 33342 (Molecular Probes). DAB staining was processed with VECTASTAIN Elite ABC Kit (PK-6200, vector laboratories, Burlingame, CA) according to manufacturer’s instructions. The images were acquired using AxioPlan-2 microscope and LSM510 confocal microscope with CCD camera.

**Immunoblot analysis** Cells were lysed with 2 x sodium dodecyl sulphate -polyacrylamide gel electrophoresis (SDS-PAGE) lysis buffer (20 mM Tris, pH 8.0, 2% SDS, 2 mM dithiothreitol (DTT), 1 mM Na_3_VO_4_, 2 mM ethylenediaminetetraacetic acid (EDTA), 20% glycerol) and sonicated. Protein concentration of each sample was determined using a micro-BCA protein assay reagent (Pierce Chemical Co., Rockford, IL) as described by the manufacturer, and equal amounts of protein were separated by SDS-PAGE and transferred to polyvinylidene difluoride (PVDF) membranes. The membranes were blocked in TBS-T (10 mM Tris, pH 8.0, 150 mM NaCl, and 0.1% Tween 20) containing 5% non-fat dried milk. The membranes were then incubated with the primary antibody at 4^o^C overnight, washed three times with TBS-T, incubated with horseradish peroxidase (HRP)-conjugated goat anti-mouse IgG or goat anti-rabbit IgG secondary antibodies for 1hr at room temperature, and then washed with TBS-T three times. The immune complexes were visualized using the enhanced chemiluminescence method. Densitometry analysis of immunoblotting images were performed using Image J software (NIH, Bethesda, MD).

**RNA extraction and quantitative real-time RT-PCR.** RNA was extracted using Trizol (Invitrogen, Carlsbad, CA) and cDNA was synthesized using iScript cDNA synthesis kit (Bio-Rad laboratories, Hercules, CA) according to the manufacturer’s directions. Quantitative real-time PCR was performed in Lightcycler (Roche Diagnostics, Germany) using Rotor-Gene SYBR Green PCR Kit (Qiagen, Hamburg, Germany). Primers of EGFR (P187403) and β2-microglobulin (B2M, 110309) were purchased from Bioneer. All reactions were performed in triplicates, and the relative transcript abundance of each tested gene was normalized to the expression levels of β2-microglobulin.

**Flow cytometry analysis** Cells were harvested, fixed with 70% ethanol, and stained with propidium iodide (PI) staining solution (20 μg/ml PI, 0.1% Sodium citrate, 50 μg/ml RNase A, 0.03% NP-40, PBS), and then analyzed by flow cytometry. The data were analyzed with Cell Quest Software (BD Bioscience, San Jose, CA).

**Annexin V/PI staining** For detection of apoptotic cells, cells were harvested and incubated for 10 min at room temperature with fluorescein isothiocyanate (FITC)-conjugated annexin V reagent and PI in binding buffer as described by the manufacturer using FITC annexin V apoptosis detection kit I (BD Biosciences, San Jose, CA), and then analyzed by flow cytometry. The data were analyzed with Cell Quest Software (BD Bioscience, San Jose, CA).

**Statistical analysis** All data points represented the mean value of experiments in triplicates. Statistical significance was determined by student’s two tailed t-test, with *p* < 0.05 taken to show significant differences between means

**References**

1. Minegishi Y, Saito M, Tsuchiya S, Tsuge I, Takada H, Hara T*, et al.* Dominant-negative mutations in the DNA-binding domain of STAT3 cause hyper-IgE syndrome. *Nature* 2007; **448**(7157)**:** 1058-1062.
2. Westerfield, M. The Zebrafish Book: a Guide for the Laboratory Use of Zebrafish (*Brachydanio erio*) University of Oregon Press, Eugene, OR 1993
3. Yabe T, Shimizu T, Muraoka O, Bae YK, Hirata T, Nojima H, et al. Ogon/Secreted Frizzled functions as a negative feedback regulator of Bmp signaling. *Development* 2003; **130**: 2705-2716
4. Bae YK, Kani S, Shimizu T, Tanabe K, Nojima H, Kimura Y, et al. Anatomy of zebrafish cerebellum and screen for mutations affecting its development. Developmental biology 2009; **330**: 406-426.

**Figure legends**

**Supplementary Figure S1.** **(a and b)** The G to A substitution in splicing acceptor site of exon 12 in REP1 genes induced zebrafish *evanescence (eva^rk10^)* mutant having abnormal stop codon by aberrant splicing. **(b)** *eva^rk10^* mutant protein lacks the C-terminal GDI domain of the wild-type REP1 protein of zebrafish. **(c)** Morphological phenotypes of WT and *eva^rk10^* mutant embryos were examined with dissecting microscope at 5 day post fertilization (dpf). Arrows indicate abnormal morphology of head, pharyngeal arch, swim bladder (black), and eye (red). **(d)** Apoptosis was visualized by TUNEL assay in WT and *eva^rk10^* mutant embryos at 5 dpf. **(e)** Paraffin-embedded zebrafish tissues were subjected to immunohistochemistry using anti-active caspase3 antibody. Eye: red arrow; Tectum: black arrow; Cerebellum: arrowhead Magnification: x50; scale bar = 100 μm. Similar results were observed in two independent experiments.

**Supplementary Figure S2.** Cancer patient-derived microarrays for cervical tissue were examined for REP1 expression using an immunoperoxidase method and isotype IgG was used as a control for staining. Scale bar = 50 μm.

**Supplementary Figure S3. (a)** A431 cells were transfected with either negative control si-RNAs (siNC) or siRNA targeting REP1 (siREP1) for 48hrs and stained with annexin V-FITC and PI, followed by flow cytometry analysis as described in “**Materials and Methods**”. Cells that were positively stained by annexin V-FITC were quantitated and considered as apoptotic cells. **(b)** A431, A549, and HT-29 cells were transfected with either siNC or siREP1 and subjected to Sub-G1 analysis by flow cytometry with error bars representing standard deviations (* *p* < 0.05). Similar results were observed in three independent experiments.

**Supplementary Figure S4.** A431, A549, and HT-29 cells were transfected with either siNC or siREP1 for 48hr and cell lysates were subjected to immunoblot analysis using indicated antibodies. Similar results were observed in two independent experiments.

**Supplementary Figure S5.** A431 cells were transfected with either siNC or two different si-RNAs specific for REP1 (siREP1-1 and siREP1-2) for 48hr. Cell lysates were subjected to immunoblot analysis using indicated antibodies. Similar results were observed in two independent experiments.

**Supplementary Figure S6. (a)** A549 cells were transfected with either empty vector (EV) and siNC, EV and siREP1, EGFR plasmid and siNC, or EGFR plasmid and siREP1 together for 48hr. Cell lysates were subjected to immunoblot analysis using indicated antibodies. **(b)** A549 cells were transfected with either siNC or siREP1 for 48hr and cell lysates were subjected to immunoblot analysis using indicated antibodies. Similar results were observed in two independent experiments.

**Supplementary Figure S7. (a)** A431 Cells were treated with 5 μM AG1478 for 24hr and cell growth was determined by MTS assay (versus control, ** *p* < 0.01). **(b and c)** A431 cells were transfected either with siNC, siEGFR, siSTAT3, or siREP1 only for 48hr. Cell lysates were subjected to immunoblot analysis using indicated antibodies **(b)** and cell growth was determined by MTS assay. (versus siNC, * *p* < 0.05) **(c)**. Similar results were observed in three independent experiments.

**Supplementary Figure S8. (a and b)** H2030 and H1975 cells were transfected with either siNC or siREP1 and subjected to Sub-G1 **(a)** and G1 phase analysis **(b)** by flow cytometry with error bars representing standard deviations (* *p* < 0.05). Similar results were observed in three independent experiments.

**Supplementary Figure S9. (a and b)** A431 cells were transfected with either EV or REP1-myc plasmid, followed by MTS assay, with error bars representing standard deviations (* *p* < 0.05) **(a)**. A431 cells transfected with REP1-myc plasmid in the **(a)** were transfected with siNC, siEGFR, or siSTAT3 for 48hrs, followed by MTS assay, with error bars representing standard deviations (* *p* < 0.05, ** *p* < 0.01) **(b)**. **(c and d)** BEAS-2B cells were transfected with either EV or REP1-myc plasmid, followed by MTS assay, with error bars representing standard deviations (* *p* < 0.05) **(c)**. BEAS-2B cells transfected with REP1-myc plasmid in the **(c)** were transfected with siNC, siEGFR, or siSTAT3 for 48hrs, followed by MTS assay, with error bars representing standard deviations (* *p* < 0.05, ** *p* < 0.01) **(d)**. Similar results were observed in three independent experiments.

**Supplementary Figure S10. (a)** A431 and B82L cell lysates were subjected to immunoblot analysis using anti- EGFR and -β-actin antibodies. **(b, c, and d)** B82L cells were transfected with either non-specific control si-RNAs (siNC) or two different si-RNAs specific for mouse REP1 (simREP1-1 and simREP1-2) and cell lysates were subjected to immunoblot analysis using anti-REP1 and -β-actin antibodies **(b)**. Cell images were taken using phase contrast microscopy. Magnification: x 100; scale bar = 100 μm **(c)**. Cell growth was measured by MTS assay at 48hr after transfection, with error bars representing standard deviations (NS: no significance) **(d).** Similar results were observed in three independent experiments.

**Supplementary Figure S11.** Tumor tissues from mice in the “Figure 6e” were processed for immunoblot analysis using indicated antibodies. Similar results were observed in two independent experiments.

**Supplementary Figure S12. (a)** Zebrafish frozen tissue sections were subjected to immunofluorescence assay using anti-EGFR antibody. Pharyngeal arch: white arrows; Esophagus: green arrow; EGFR: red; Nucleus; blue**.** Magnification: x50, Scale bar = 100 μm**.** **(b)** Paraffin-embedded zebrafish tissues were subjected to immunohistochemistry using anti-EGFR antibody. Scale bar = 100 μm**.** **(c)** Cell lysates from WT and *eva^rk10^* mutant embryos at 5dpf were processed for immunoblot analysis using anti-EGFR and β-actin antibodies. β-actin was used as a loading control. Similar results were observed in two independent experiments.

**Supplementary Figure S13. (a)** Kaplan-Meier curve showed probability of patients with gastric tumor expressing high or low level of REP1. **(b)** AGS and SNU638 cells were transfected with either siNC or siREP1 for 48hr and cell lysates were suggested to immunoblot analysis using indicated antibodies. Similar results were observed in three independent experiments.

**Supplementary Figure S14. (a)** A431 cells were transfected with either siNC or siREP1 for 30hr and total RNA was extracted. The levels of EGFR mRNA were determined by RT-qPCR (NS: no significant differences). **(b)** After 29hr transfection of A431 cells with either siNC or siREP1, cells were further treated with 5 μM MG132 for 1hr, followed by immunoblot analysis using anti-HIF1α, -EGFR, and -β-actin antibodies. The levels of EGFR were quantified by densitometry and normalized to β-actin levels. **(c)** After 6hr transfection of A431 cells with either siNC or siREP1, cells were further treated with 10 μM chloroquine for 24hr, followed by immunoblot analysis using anti-EGFR, -REP1, and -β-actin antibodies. The levels of EGFR were quantified by densitometry and normalized to β-actin levels. The experiments were performed three times with similar results.

**Supplementary Figure S15. (a)** A431 cells were transfected with either siNC or siREP1 for 48hr and cell lysates were subjected to immunoblot analysis using anti-phospho-EGFR (Y1045) and -β-actin antibodies. Similar results were observed in two independent experiments.

**Supplementary Figure S16.** A431 cells were transfected with either siNC or siREP1 for 48hr and cell lysates were subjected to immunoblot analysis using anti-REP1, -EGFR, -Rab5, -Rab7, and -Rab11 antibodies. Similar results were observed in three independent experiments.
