# Supplementary material for: Oncogenic role of rab escort protein 1 through EGFR and STAT3 pathway
Source: Cell Death Dis. 2017 Feb 23;8(2):e2621–. doi: 10.1038/cddis.2017.50 (PMC5386492; doi:10.1038/cddis.2017.50)
Supplement: Supplementary Tables [file cddis201750x2.docx]

Supplementary Table S1. Summary of REP1 expression and clinicopathological feature in cervical cancer

| Patient character | REP1 expression | | | *p*-value |
| --- | --- | --- | --- | --- |
|  | Number | -/+ | ++/+++ |  |
| **Sex** |  |  |  |  |
| Male | 0 | 0 (0.0%) | 0 (0.0%) |  |
| Female | 50 | 18 (36.0%)+ | 32 (64.0%) |  |
| **Age** |  |  |  | 0.3784 |
| ≤57 | 35 | 14 (40.0%) | 21 (60.0%) |  |
| >57 | 15 | 4 (26.7%) | 11 (73.3%) |  |
| **Clinical stage** |  |  |  | 0.5726 |
| 0 | 3 | 1 (33.3%) | 2 (66.7%) |  |
| I | 27 | 9 (33.3%) | 18 (66.7%) |  |
| II | 1 | 0 (0.0%) | 1 (100.0%) |  |
| III | 19 | 8 (42.1%) | 11 (57.9%) |  |
| **N Classification** |  |  |  | 0.7556 |
| N_0_ | 32 | 11 (34.4%) | 21 (65.6%) |  |
| N_1_ | 18 | 7 (38.9%) | 11 (61.1%) |  |
| N_2_ | 0 | 0 (0.0%) | 0 (0.0%) |  |
| **T Classification** |  |  |  | 0.4895 |
| T_1_ | 42 | 16 (38.1%) | 26 (61.9%) |  |
| T_2_ | 8 | 2 (25.0%) | 6 (75.0%) |  |
| T_3_ | 0 | 0 (0.0%) | 0 (0.0%) |  |
| T_4_ | 0 | 0 (0.0%) | 0 (0.0%) |  |
| **Histology** |  |  |  |  |
| Adenocarcinoma | 2 | 2 (100.0%) | 0 (0.0%) | 0.6329 |
| Squamous carcinoma | 47 | 16 (34.0%) | 31 (66.0%) |  |
| Others | 1 | 0 (0.0%) | 1 (100.0%) |  |

Supplementary Table S2. Summary of REP1 expression and clinicopathological feature in lung cancer

| Patient character | REP1 expression | | | *p*-value |
| --- | --- | --- | --- | --- |
|  | Number | -/+ | ++/+++ |  |
| **Sex** |  |  |  | 0.5744 |
| Male | 26 | 8 (30.8%) | 18 (69.2%) |  |
| Female | 14 | 5 (35.7%) | 9 (64.3%) |  |
| **Age** |  |  |  | 0.8409 |
| ≤57 | 21 | 6 (28.6%) | 15 (71.4%) |  |
| >57 | 19 | 7 (36.8%) | 12 (63.2%) |  |
| **Clinical stage** |  |  |  | 0.7834 |
| I | 13 | 5 (38.5%) | 8 (61.5%) |  |
| II | 14 | 3 (21.4%) | 11 (78.6%) |  |
| III | 12 | 4 (33.3%) | 8 (66.7%) |  |
| IV | 1 | 1 (100.0%) | 0 (0.0%) |  |
| **N Classification** |  |  |  | 0.6753 |
| N_0_ | 19 | 7 (36.8%) | 12 (63.2%) |  |
| N_1_ | 12 | 4 (33.3%) | 8 (66.7%) |  |
| N_2_ | 9 | 2 (22.2%) | 7 (77.8%) |  |
| **T Classification** |  |  |  | 0.4735 |
| T_1_ | 3 | 1 (33.3%) | 2 (66.7%) |  |
| T_2_ | 31 | 9 (29.0%) | 22 (71.0%) |  |
| T_3_ | 1 | 1 (100.0%) | 0 (0.0%) |  |
| T_4_ | 5 | 2 (40.0%) | 3 (60.0%) |  |
| **Histology** |  |  |  | 0.2851 |
| Adenocarcinoma | 8 | 1 (14.3%) | 7 (85.7%) |  |
| Squamous carcinoma | 13 | 6 (46.2%) | 7 (53.8%) |  |
| Others | 19 | 5 (26.3%) | 14 (73.7%) |  |

Supplementary Table S3. Summary of REP1 expression and clinicopathological feature in colorectal cancer

| Patient character | REP1 expression | | | *p*-value |
| --- | --- | --- | --- | --- |
|  | Number | -/+ | ++/+++ |  |
| **Sex** |  |  |  | 0.4385 |
| Male | 28 | 6 (21.4%) | 22 (78.6%) |  |
| Female | 12 | 4 (33.3%) | 8 (66.7%) |  |
| **Age** |  |  |  | 0.4780 |
| ≤57 | 20 | 4 (20.0%) | 16 (80.0%) |  |
| >57 | 20 | 6 (30.0%) | 14 (70.0%) |  |
| **Clinical stage** |  |  |  | 0.9197 |
| I | 2 | 0 (0.0%) | 2 (100.0%) |  |
| II | 11 | 2 (18.2%) | 9 (81.8%) |  |
| III | 15 | 7 (46.7%) | 8 (53.3%) |  |
| IV | 12 | 1 (8.3%) | 11 (91.7%) |  |
| **N Classification** |  |  |  | 0.1325 |
| N_0_ | 15 | 2 (13.3%) | 13 (86.7%) |  |
| N_1_ | 12 | 3 (25.0%) | 9 (75.0%) |  |
| N_2_ | 13 | 5 (38.5%) | 8 (61.5%) |  |
| **T Classification** |  |  |  | 0.3586 |
| T_1_ | 0 | 0 (0.0%) | 0 (0.0%) |  |
| T_2_ | 3 | 0 (0.0%) | 3 (100.0%) |  |
| T_3_ | 34 | 9 (26.5%) | 25 (73.5%) |  |
| T_4_ | 3 | 1 (33.3%) | 2 (66.7%) |  |
| **Histology** |  |  |  | 0.0832 |
| Adenocarcinoma | 39 | 9 (23.1%) | 30 (76.9 %) |  |
| Squamous carcinoma | 0 | 0 (0.0%) | 0 (0.0%) |  |
| Others | 1 | 1 (100%) | 0 (0.0%) |  |
