# Supplementary material for: Oncogenic role of rab escort protein 1 through EGFR and STAT3 pathway
Source: Cell Death Dis. 2017 Feb 23;8(2):e2621–. doi: 10.1038/cddis.2017.50 (PMC5386492; doi:10.1038/cddis.2017.50)

Supplementary Figure S1

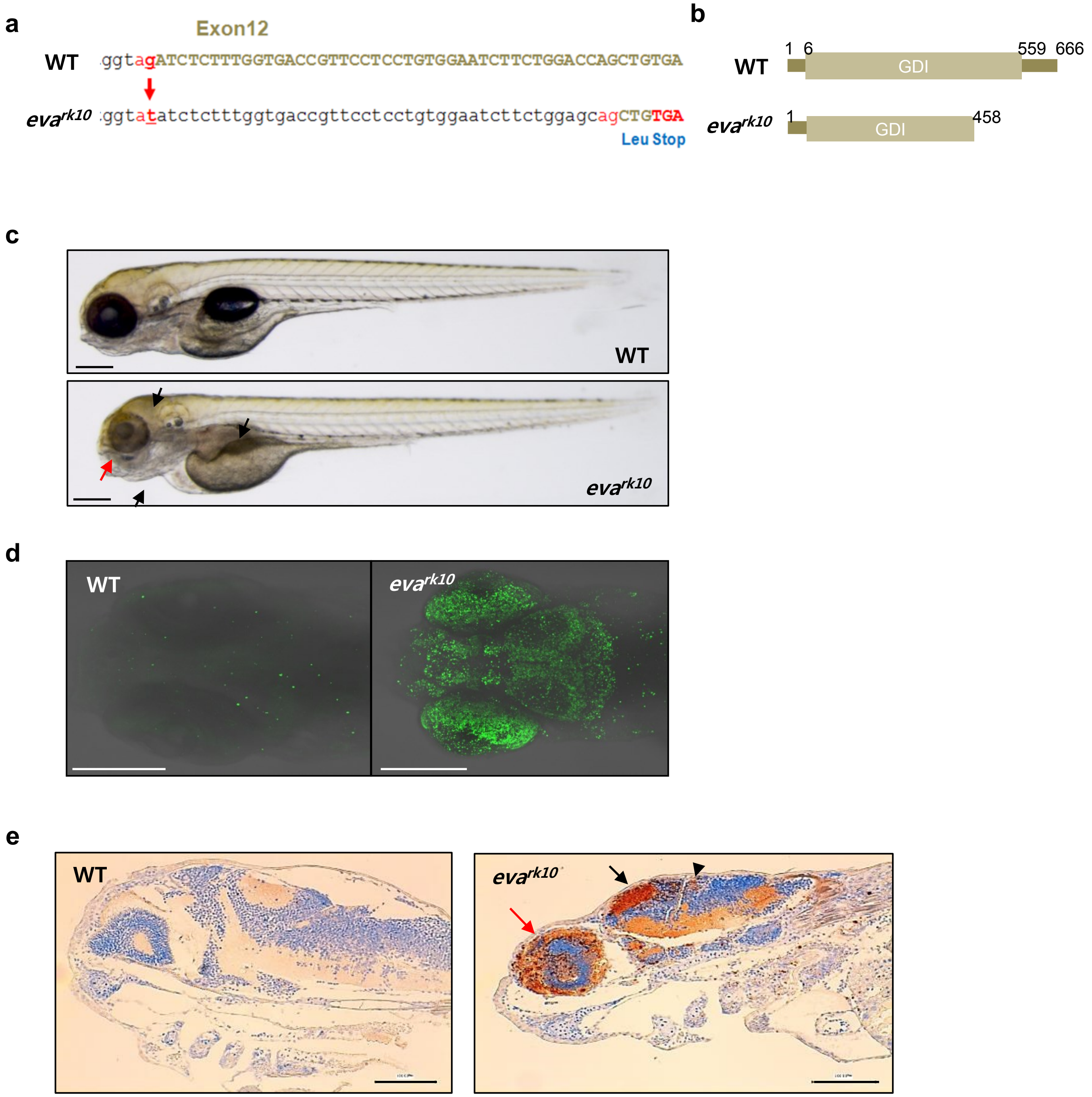

Supplementary Figure S2

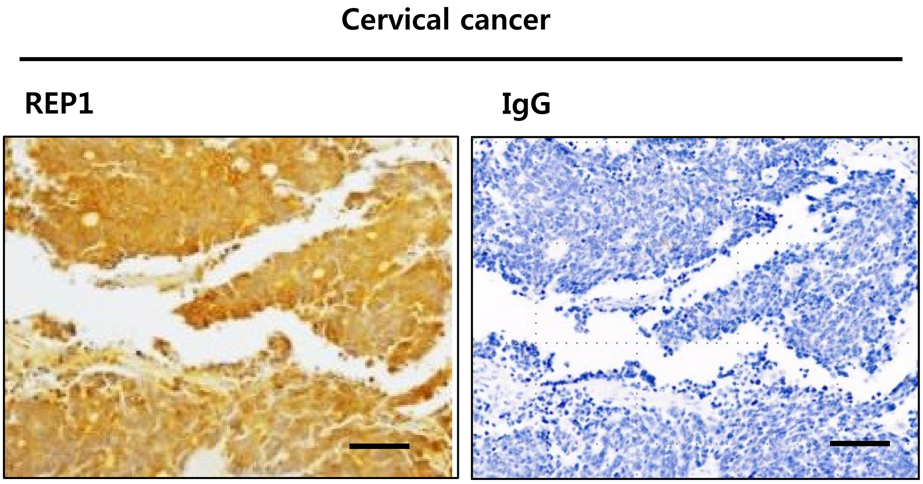

Supplementary Figure S3

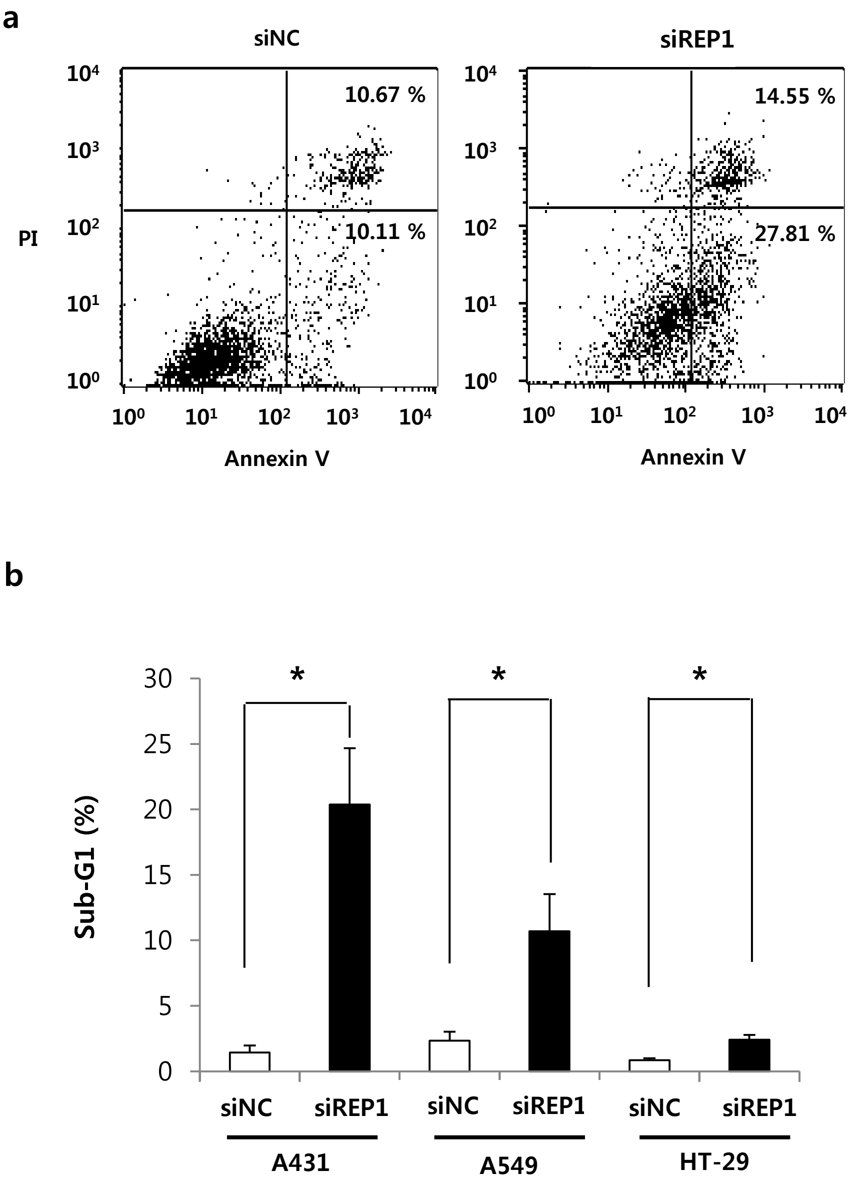

Supplementary Figure S4

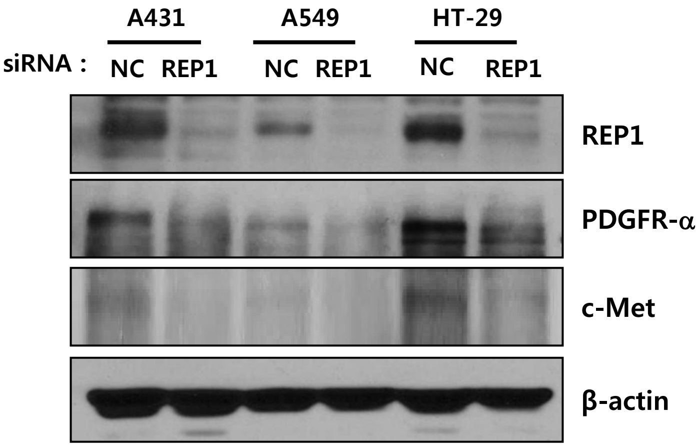

Supplementary Figure S5

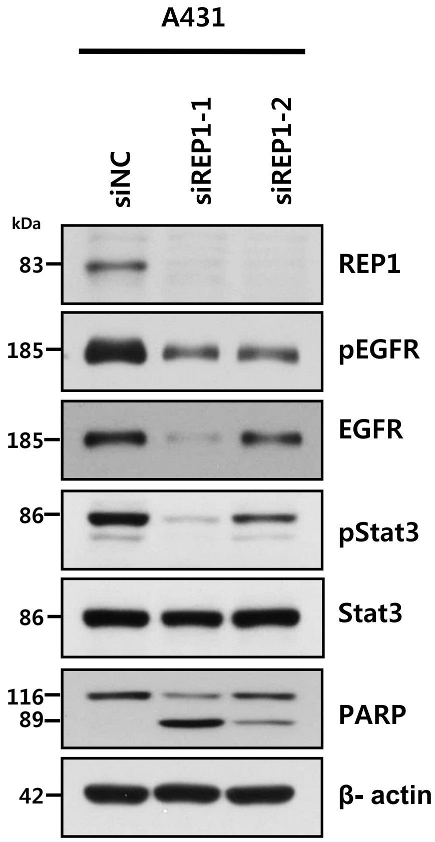

Supplementary Figure S6

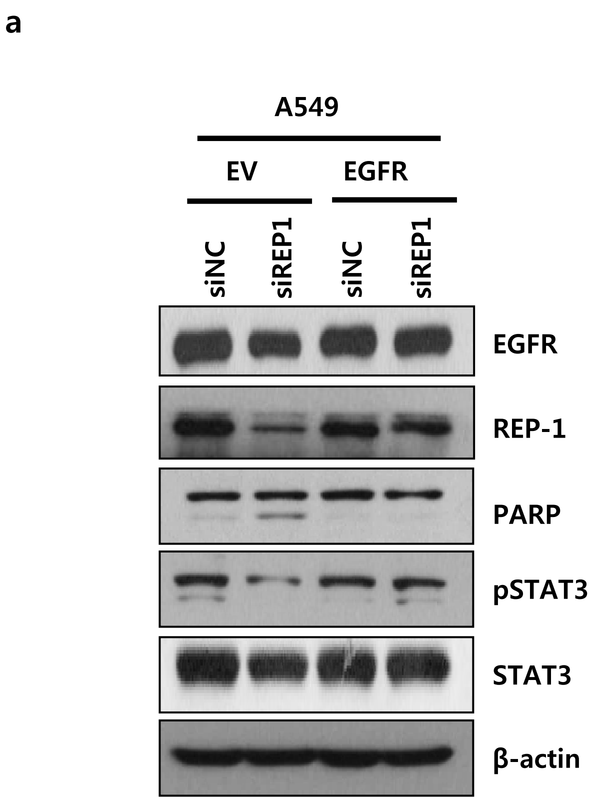

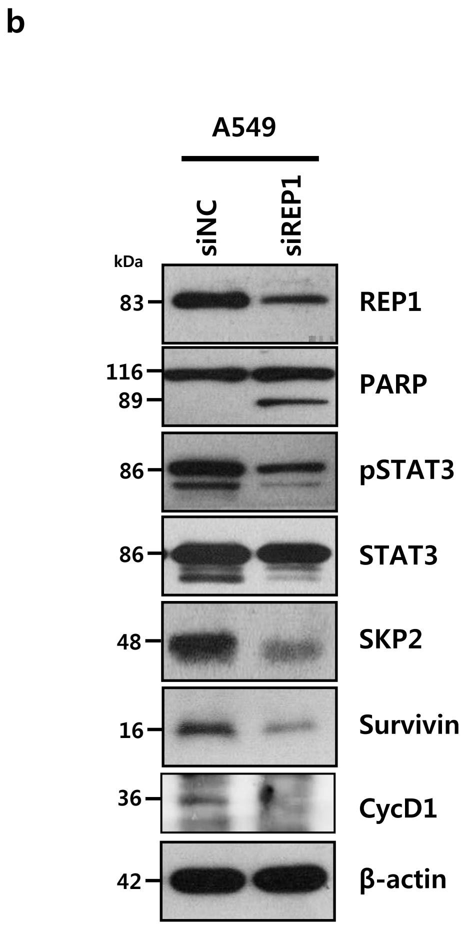

Supplementary Figure S7

a

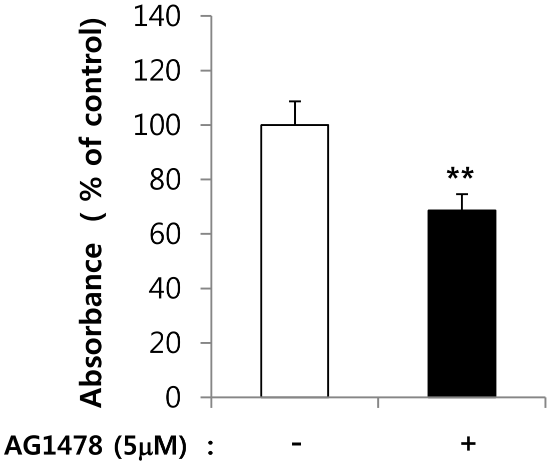

b

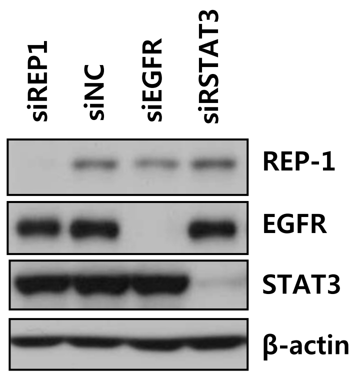

c

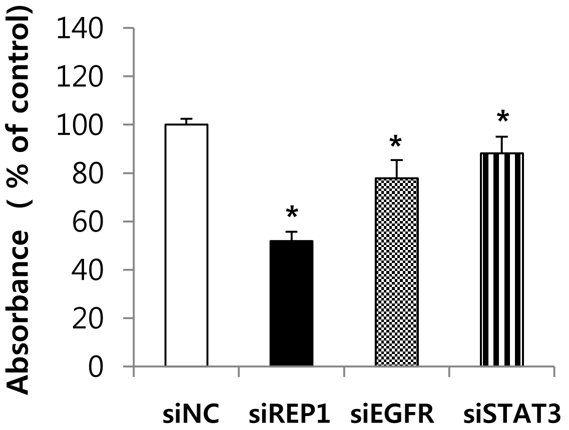

Supplementary Figure S8

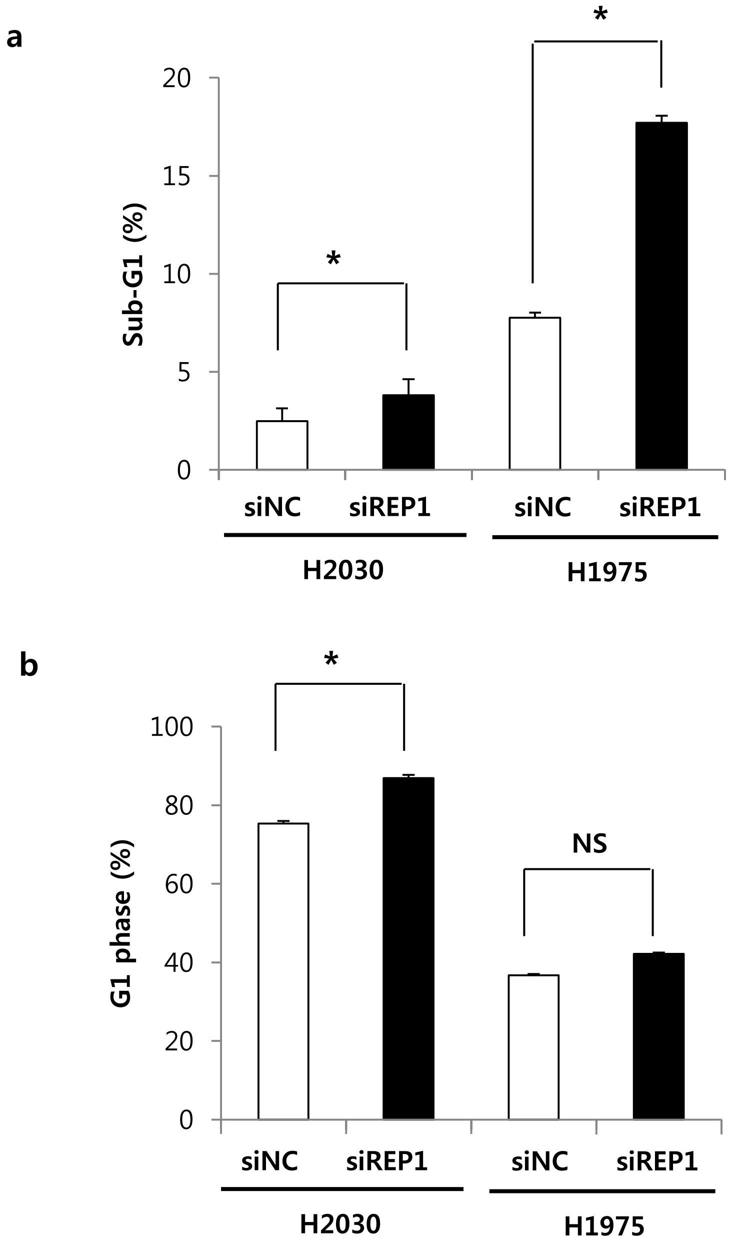

Supplementary Figure S9

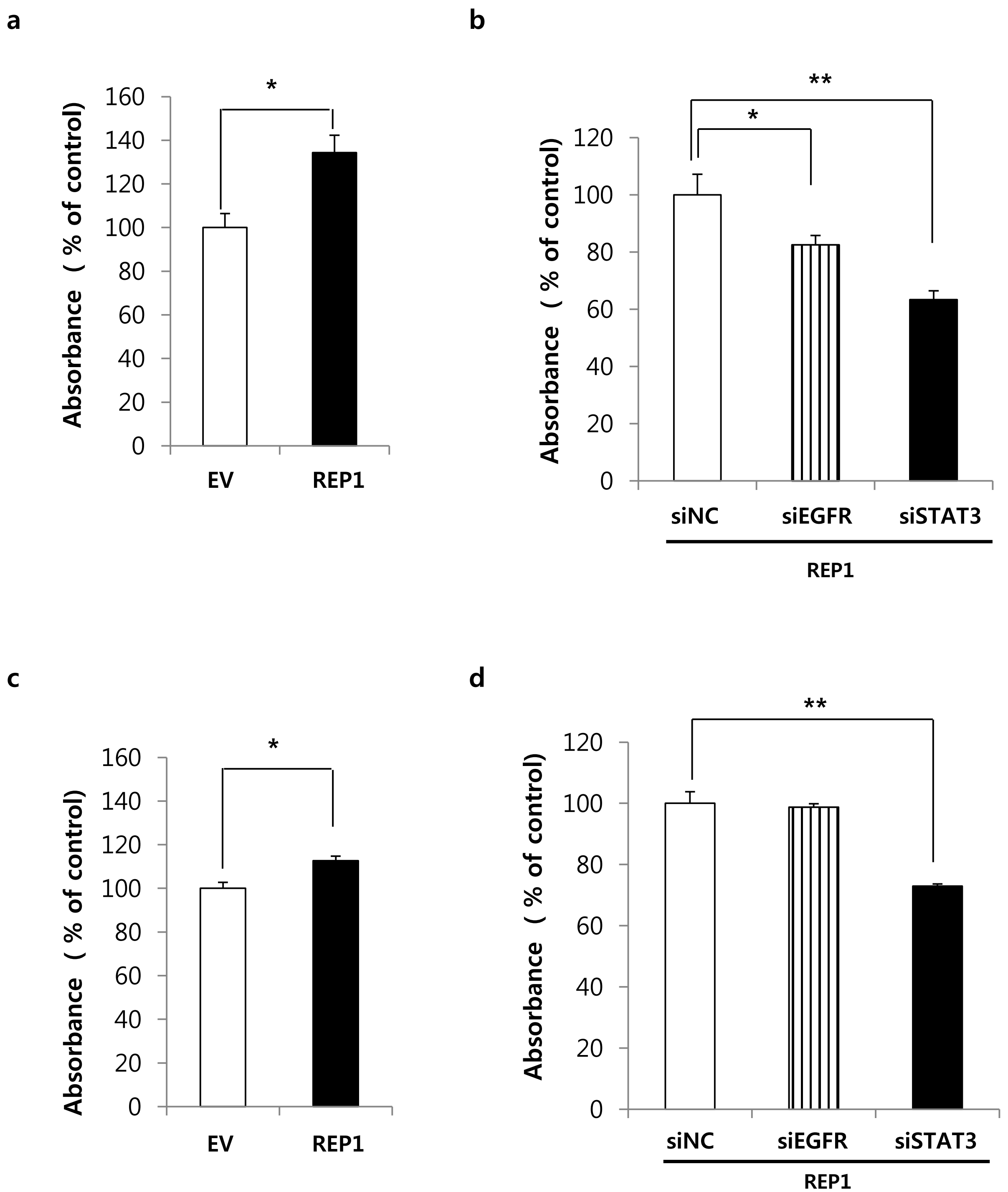

Supplementary Figure S10

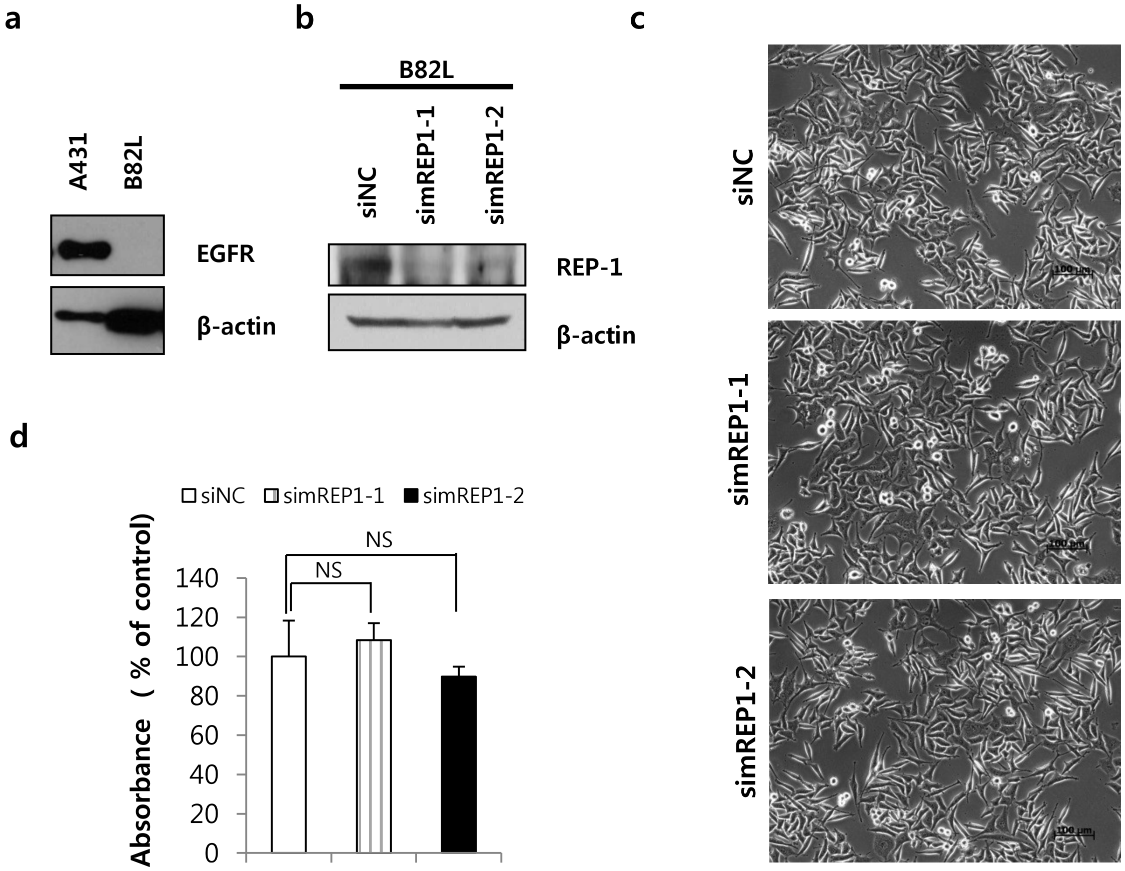

Supplementary Figure S11

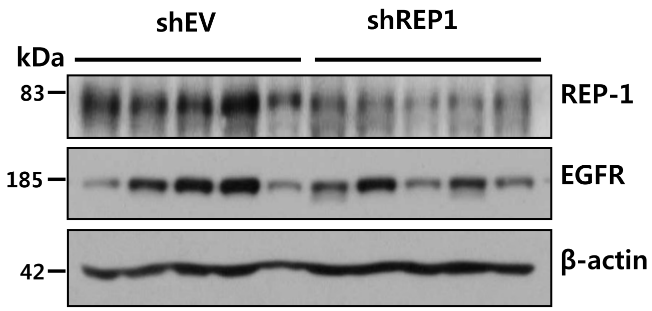

Supplementary Figure S12

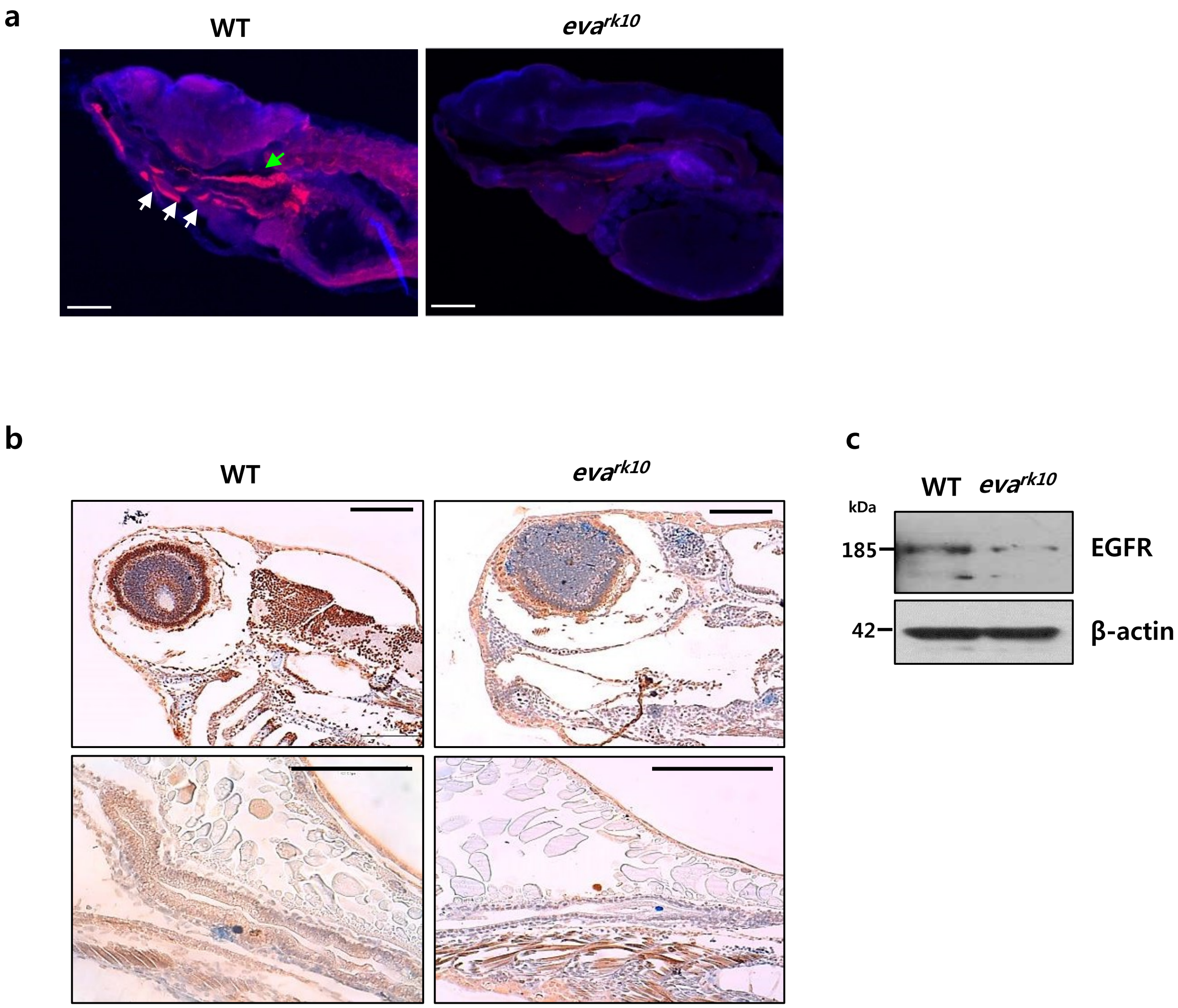

Supplementary Figure S13

a

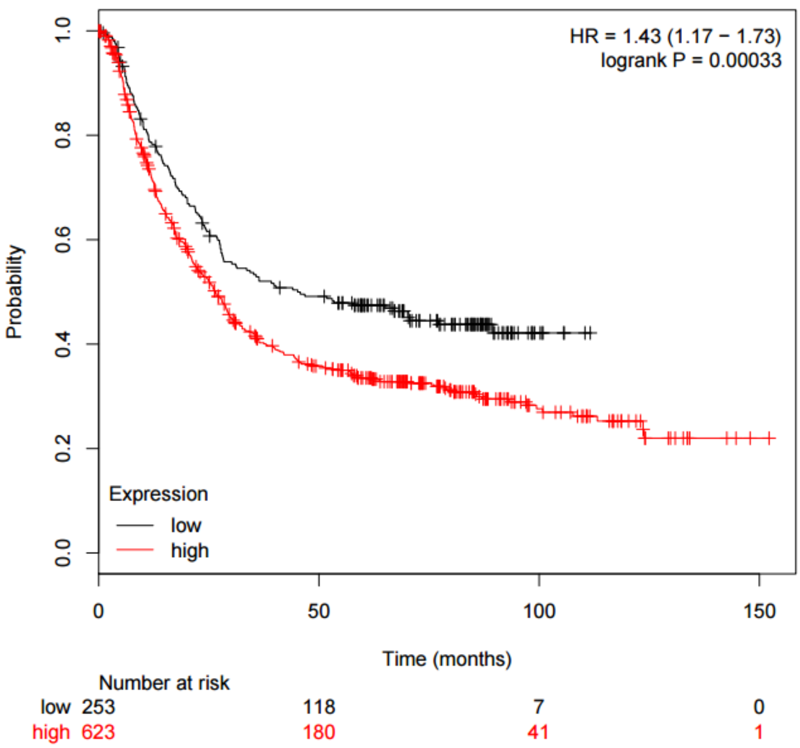

b

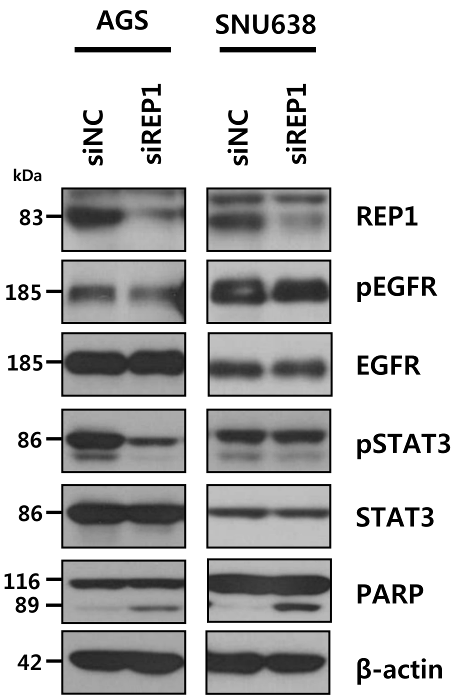

Supplementary Figure S14

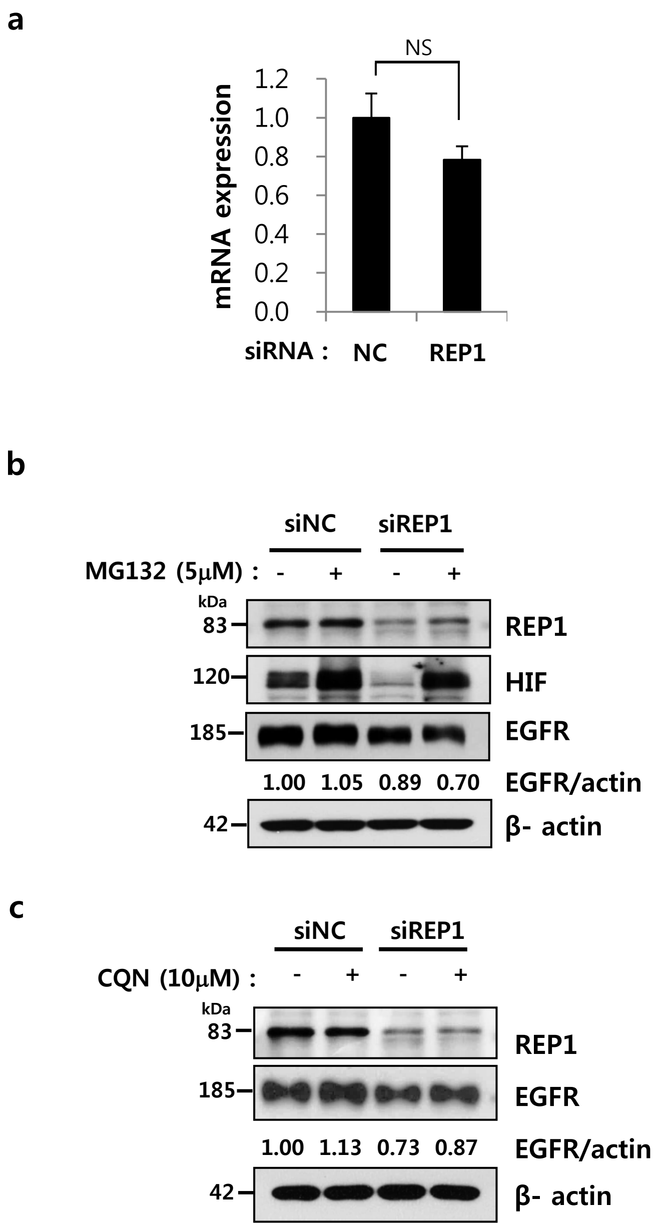

Supplementary Figure S15

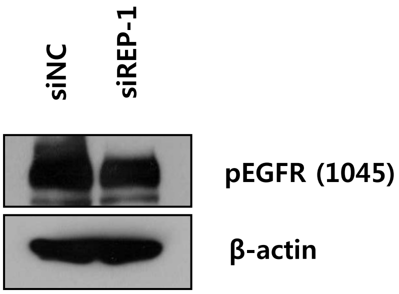

Supplementary Figure S16

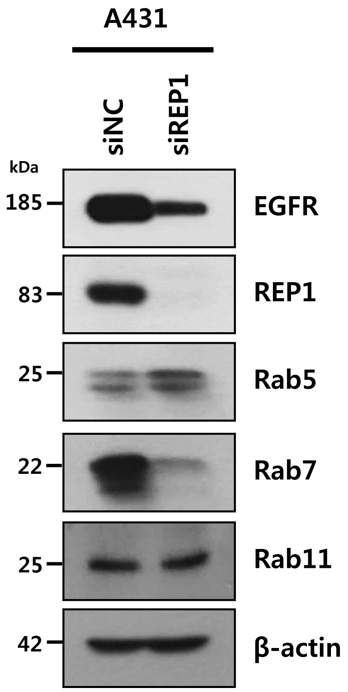

Supplement: Supplementary Figures [file cddis201750x3.pdf]
